# The efficacy and safety of perioperative glucocorticoid for total knee arthroplasty: a systematic review and meta-analysis

**DOI:** 10.1186/s12871-024-02530-9

**Published:** 2024-04-15

**Authors:** Fangyan Liu, Mei Duan, Huiqun Fu, Tianlong Wang

**Affiliations:** 1 Department of Anesthesiology, Xuanwu Hospital, Capital Medical University, 45 Changchun Street, Xicheng District, Beijing, 100053 China; 2 National Clinical Research Center for Geriatric Diseases, Beijing, China; 3 Beijing Institute for Brain Disorders, Beijing, China; 4 Center for Sleep and Consciousness Disorders, Collaborative Innovation Center for Brain Disorders, Beijing Institute of Brain Disorders, Capital Medical University, Beijing, China

**Keywords:** TKA, Glucocorticoid, VAS, Inflammation, Meta-analysis

## Abstract

**Background:**

An increasing number of individuals undergo total knee arthroplasty (TKA), which can result in pain, limited motor function and adverse complications such as infection, nausea and vomiting. Glucocorticoids have been shown anti-inflammatory and antiemetic effects, but can also elevate blood glucose levels and increase the risk of wound infection. Thus, it is essential to investigate the efficacy and safety of glucocorticoid usage in TKA.

**Method:**

A comprehensive systematic search of PubMed, Medline, EMBASE, Cochrane databases, to identify relevant randomized controlled trials (RCTs) of glucocorticoid application in TKA. The primary outcomes assessed were the postoperative pain assessment. Secondary outcomes included the range of motion in knee joint, levels of inflammatory cytokines, adverse complications, and the length of hospital stay.

**Results:**

Thirty-six randomized controlled trials were included in the final analysis. The glucocorticoid group exhibited significant reduction in the resting VAS scores on postoperative days 1, 2 (POD1, 2)and postoperative 3 months (POM3), as well as decreased morphine consumption on POD1 and increased range of motion (ROM) in knee joint on POD1, 3. Additionally, the glucocorticoid group exhibited decreased levels of postoperative inflammatory cytokines and the incidence of PONV along with a shorter length of hospital stay. The blood glucose concentration was significantly increased in the glucocorticoid group on POD1 compared with the control group. While the blood glucose on POD2 and occurrence of postoperative adverse complications were similar between two groups including wound infection and venous thrombosis. The periarticular injection analgesia (PIA) group demonstrated lower VAS scores on POD2 comparing to the systemic administration (SA) group according to two studies. However, there was no significant difference of the resting VAS on POD1 and POD2 between PIA and SA group across all studies.

**Conclusion:**

Perioperative glucocorticoids treatment in TKA significantly reduced short-term pain score and opioid-use which was probably not patient relevant. The application of glucocorticoids in TKA implied a beneficial trend in analgesic, anti-inflammatory, and antiemetic effects, as well as improved range of motion and shortened hospital stay. While it will not increase the risk of continued high glucose, postoperative wound infection and venous thrombosis.

**Supplementary Information:**

The online version contains supplementary material available at 10.1186/s12871-024-02530-9.

## Introduction

Total knee arthroplasty (TKA) is a highly effective surgical treatment for severe knee arthritis which could reduce pain and maintain motor function [1, 2]. However, many patients still experience moderate to severe pain following TKA and require increased analgesic use postoperatively [3]. Various analgesic methods, including nerve blocks, local anesthetic infiltration, and intravenous opioids, are utilized in these patients [4–6]. Unfortunately, patients often report dissatisfaction with pain management and are prone to complications such as nausea and vomiting [7]. Besides, surgical intervention triggers a local inflammatory response, which further leads to systemic inflammatioty response through the release of inflammatory factors [8]. Inflammation can further exacerbate postoperative joint pain, restrict knee joint range of motion, impede rapid postoperative rehabilitation, prolong hospital stay [9].

Glucocorticoids are commonly employed in TKA surgery due to their anti-inflammatory and antiemetic properties [10]. Some studies have indicated that glucocorticoids can effectively alleviate postoperative pain, reduce opioid consumption and mitigate complications such as nausea and vomiting in TKA patients. However, other studies have raised concerns about glucocorticoids usage, including potential adverse effects such as elevated blood glucose levels, infection, and impaired wound healing, particularly in patients receiving long-term glucocorticoid therapy [11]. Therefore, further investigation is required to determine the efficacy and safety of glucocorticoids in total knee arthroplasty. Additionally, the optimal medication, types, and number of doses of glucocorticoids administered perioperatively in TKA remain uncertain. The objective of this systematic review is to evaluate the available randomized controlled trials that investigate the efficacy and safety associated with glucocorticoid use in total knee arthroplasty. The main purposes of this paper include the following: (1) To explore the effect of glucocorticoids on postoperative rest pain and morphine consumption in patients after TKA; (2) To explore the effect of glucocorticoids on range of knee’s motion and length of hospital stay in patients after TKA; (3) To explore the effect of glucocorticoids on peripheral inflammation in patients after TKA; (4) To explore the adverse complications of glucocorticoids in patients after TKA, including PONV, high blood glucose, wound infection and venous thrombosis. Meanwhile, to investigate the impact of glucocorticoid dosage on PONV and compare the effect of the glucocorticoid by periarticular injection and system administration.

## Materials and methods

This systematic review and meta-analysis was conducted according to the PRISMA (Preferred Reporting Items for Systematic Reviews and Meta-Analyses) guidelines [12]. And this systemic review has been registered (PROSPERO ID: CRD42023435063).

### Literature search and selection of studies

PubMed, Medline, EMBASE, Cochrane databases, were searched to identify relevant studies from inception to January 22, 2022. To include all the articles on steroid supplementation for TKA, the string used for the literature search included all the synonyms of TKA and the different drugs that could be supplemented with the following search terms (total knee arthroplasty OR TKA OR total knee replacement OR TKR) AND (Dexamethasone OR steroid OR glucocorticoid OR corticosteroid OR Methylprednisolone OR betamethasone OR cortisone OR hydrocortisone OR hexadecadrol OR prednisolone OR triamcinolone) without putting restrictions on language. After the removal of duplicates, all the records collected were then screened by title and abstract and, when necessary, by reading the full text article. The entire process was completed independently by two authors (LFY, DM). We excluded conference abstracts and case reports unless they had subsequently been published as full articles.

Studies were selected based on the following inclusion criteria: 1) randomized clinical trials; 2) comparison of two or more glucocorticoid interventions in primary TKA; 3) postoperative evaluation included VAS, morphine consumption, inflammatory response (C-reactive protein and Interleukin-6), ROM, adverse effects and LOS. The exclusion criteria were: 1) studies included knee and hip arthroplasty without discrete data for TKA; 2) overall sample size fewer than ten patients; 3) unicompartmental knee arthroplasty (UKA) or revision of total knee arthroplasty.

### Data extraction

Two reviewers independently extracted data, and the third reviewer checked the consistency between them. A standard form was used; the extracted items included author, study design, sample size, publishing date, country, case number, age, gender, intervention method, dosages and type of anesthesia and follow-up term. The primary outcome was the analgesic effect, including resting VAS on the 1st, 2nd day and 3rd month after TKA and morphine consumption on the 1st day after operation. Secondary outcome measures included inflammatory response (C-reactive protein and Interleukin-6), ROM during the 3 days postoperatively, adverse effects and LOS.

### Assessment of methodological quality

Two reviewers (LFY, DM) independently assessed the methodological quality of the included studies which were performed by the Cochrane Collaboration for Systematic Reviews, including assessment of random sequence generation, allocation sequence concealment, blinding of participants and personnel, blinding of outcomes assessment, incomplete outcome data, selective reporting, and other bias. Quantitative synthesis was conducted using Review Manager (RevMan 5.3). As the guidelines set out by the GRADE (Grading of Recommendations, Assessment, Development and Evaluations), all scores for each measured outcome were converted to a common scale [13].The overall methodological quality of each included study was characterized as "low risk of bias", "high risk of bias", or "unclear risk of bias". Differences will be resolved by consensus after discussion and, if necessary, a third reviewer will be consulted.

### Statistical analysis

We used RevMan 5.3 to conduct this meta-analysis. For dichotomous data, RRs with 95% CIs were used to express the effect sizes, while mean difference and 95% CIs were used for continuous data. Firstly, we conducted a heterogeneity test to evaluate the extent of heterogeneity in combination with the I^2^-test [14]. A fixed-effects model was used to conduct the meta-analysis if no heterogeneity (*p* > 0.1 and I^2^ < 50%) was observed among the studies. If significant heterogeneity (*p* ≤ 0.1 or I^2^ ≥ 50%) was observed, then a random-effects model was used for the meta-analysis. The Z-test was used to determine the significance of the pooled effect size, and *p* < 0.05 was considered statistically significant. Publication bias was assessed using the funnel plots, Egger’s regression test [15], and Begg’s adjusted rank correlation [16], which were conducted with the Stata software (Stata Corp., TX, USA; version 15.0).

## Results

### Search results

**Fig. 1:**
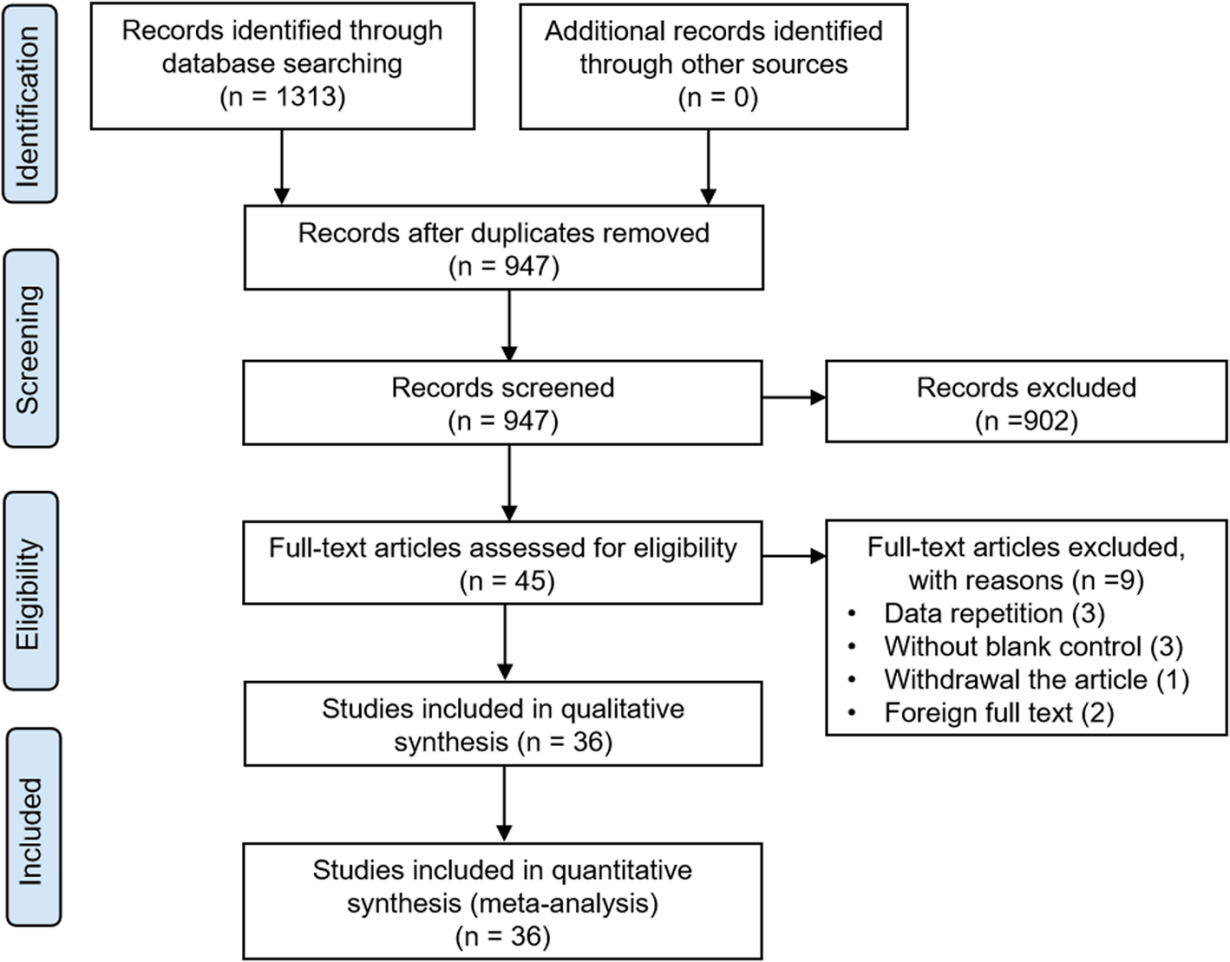
Preferred Reporting Items for Systematic Reviews and Meta-Analyses (PRISMA) diagram of study selection

In the recent study, a total of 1,313 studies were identified based on the inclusion criteria. After removing 366 duplicates, 947 records remained. From these, 902 articles were excluded based on the title and abstract screening. 36 RCTs were ultimately enrolled after browsing the whole text and 9 studies were excluded for specific reasons (Fig. 1).

**Fig. 2:**
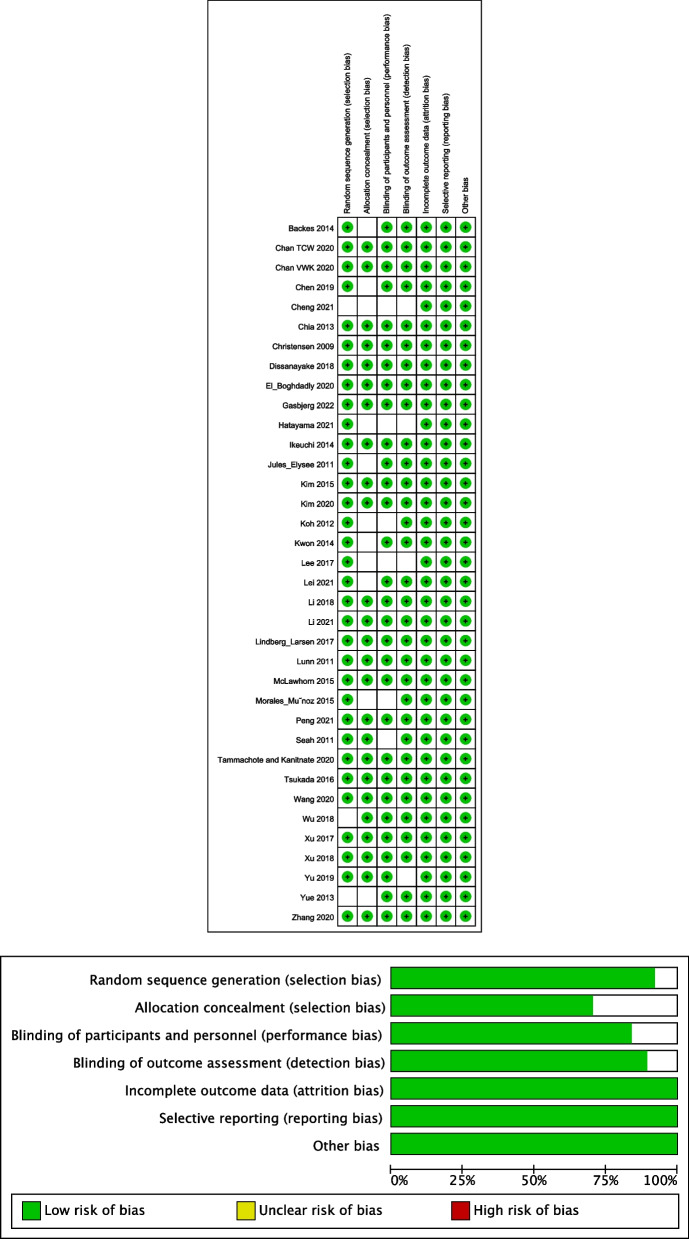
Risk of bias evaluation of included studies and summary of decisions made for each criterion

The sample size of the included studies ranged from 23 to 323 participants. Among the included studies, a total of 3,048 patients who underwent primary unilateral TKA were represented in thirty-one RCTs. Additionally, one study by Morales-Munoz et al. included 54 patients who underwent knee replacement surgery, and four studies encompassed 211 patients who underwent bilateral TKA. The follow-up period across the studies varied from 24 h to 1 year. In terms of glucocorticoid administration, 1,188 patients received systemic glucocorticoid administration either before anesthesia induction or after surgery, while 663 patients had been implemented with glucocorticoid in the cocktail protocol for intraoperative periarticular infiltration. The specific drugs utilized in the studies included dexamethasone in twenty studies, methylprednisolone in six studies, triamcinolone acetonide in five studies, hydrocortisone in three studies, betamethasone in two studies, and prednisone in one study. The basic characteristics of the studies are described in the Table 1 [17–52]. The risk of bias assessment of RCT studies is presented in Fig. 2.

**Table 1:** Characteristic of the included studies

**Study**	**Year**	**Country**	**Sample size**	**Mean age**	**Gender (M:F) (I/C)**	**Anesthesia**	**Intervention**	**Pain manage**	**Relevant outcome**	**Follow-up**
			**(I/C)**							
Gasbjerg et al. [17]	2022	Danish	161/162	69/68	80:81/74:88	S/G	24 mg DXM prior surgery	Oral paracetamol and ibuprofen prior and after surgery, LIA, PCA (morphine), Morphine for rescue analgesia.	VAS and adverse effects	3 months
Lei et al. [18]	2021	China	62/63	66.0 /66.0	12:50/8:55	G	10 mg DXM prior to induction	LIA, oral diclofenac or an IM of morphine as required.	VAS, ROM, Inflammatory cytokines and adverse effects	3 months
Cheng et al. [19]	2021	China	49/49^a^	63.7/64.8	10:45/12:43	S	Oral prednisone (10 mg qd, from pod1, for 2 weeks)	Oral celecoxib prior surgery, LIA, celecoxib on pod1 and tramadol for 2 weeks.	VAS and adverse effects	3 months
Zhang et al. [20]	2020	China	31/32	66.3/67.3	6:25/4:28	G	20 mg DXM after anesthesia, 10 mg DXM at 24 and 48 h	PIA, enteric-coated diclofenac sodium bid.	Inflammatory cytokines and adverse effects	1 year
Tammachote and Kanitnate [21].	2020	Thailand	50/50	67 /69	8:42/6:44	S	0.15 mg/kg DXM after anesthesia	PIA, IV ketorolac or morphine as required during the pod2, oral naproxen, oral tramadol as required on pod3. Extended-released acetaminophen, nortriptyline and pregabalin at bedtime.	VAS, ROM and adverse effects	3 months
Kim et al. [22]	2020	Korea	45/44	69.3 /68.2	4:41/3:41	S	10 mg DXM 1 h prior surgery	PIA, PCA (fentanyl and Acupan), oral celecoxib, tramadol/acetaminophen, oral oxycodone, IV pethidine or fentanyl patches as emergency required.	VAS and adverse effects	1 week
Chan et al. [23]	2020	China	46/45	75.5/75.3	11:35/13:32	S	8 mg DXM prior induction	LIA, PCA morphine, oral analgesic regimen (pregabalin, paracetamol and celecoxib).	VAS, ROM, Inflammatory cytokines and adverse effects	1 year
Yu et al. [24]	2019	China	45/43	65.1/64.2	19:26/17:26	G	10 mg DXM after the anesthesia and repeated at 24 h	Oral oxycodone or an IM of pethidine hydrochloride as required.	VAS, ROM, Inflammatory cytokines and adverse effects	3 months
Chen et al. [25]	2019	China	30/30	66.7/68.1	8:22/8:22	S	125 mg MP on the induction	LIA, PCA Morphine, Oral or IV medications (Gabapentin, Esomeprazole, Paracetamol, Cefazolin, Tranexamic acid, Etoricoxib, Bisacodyl, Metoclopramide, Dihydrocodeine).	Inflammatory cytokines and adverse effects	1 year
Xu et al. [26]	2018	China	60/61	64.5/65.8	11:49/8:53	G	20 mg DXM prior induction	PAI, oral diclofenac, oxycodone HCl or IM of morphine as required.	VAS, ROM, Inflammatory cytokines and adverse effects	3 months
Wu et al. [27]	2018	China	50/50	66.9/67.4	17:33/18:32	G	10 mg DXM 1 h prior surgery	MOAG from 1 day prior operation (celecoxib, pregabalin), oral oxycodone or an IM of pethidine hydrochloride as required.	VAS, ROM, Inflammatory cytokines and adverse effects	3 months
Li et al. [28]	2018	China	36/32	63.9 /64.7	7:29/5:27	NR	100 mg HC, 2 h prior and 8 h after surgery	Oral celecoxib, LIA, PCA, IM of pethidine hydrochloride as required.	VAS, ROM and adverse effects	30 days
Dissanayake et al. [29]	2018	Australia	41/40	NR	NR	S/G	8 mg DXM at induction and at 24 h	LIA, paracetamol, gabapentin for 2,3 days post-surgery, oxycodone/naloxone for 4,5 days, ibuprofen or celecoxib, oxycodone and tramadol slow release as required.	VAS and adverse effects	6 weeks
Xu et al. [30]	2018	China	54/54	63.6 /63.6	8:46/9:45	G	10 mg DXM after anesthesia, 10 mg DXM returned to the inpatient unit	PIA, MOAG from 1 day prior operation (diclofenac, pregabalin), oral oxycodone or an IM of parecoxib as required.	VAS, ROM, Inflammatory cytokines and adverse effects	3 days
Lindberg-Larsen et al. [31]	2017	Denmark	33/30	65.0/67.7	13:20/15:15	S	125 mg MP after anesthesia	Oral paracetamol, naproxen 1 h prior surgery, LIA, oral paracetamol and naproxen. Opioids as request.	VAS, Inflammatory cytokines and adverse effects	24 h
Lee et al. [32]	2018	Canada	60/60	64.6/67	18:42/23:37	S	8 mg DXM over 10 min after block	Oral acetaminophen before surgery, LIA, PCA hydromorphone, oral acetaminophen, oral opioids (hydromorphone or oxycodone), IV ketorolac or subcutaneous opioids as needed,	VAS and adverse effects	48 h
Morales-Munoz et al. [33]	2015	Spain	27/27^b^	68.8/68.8	8:19/6:21	S	8 mg DXM	0.5% ropivacaine (20 ml) for FNB	VAS and adverse effects	48 h
McLawhorn et al. [34]	2015	America	11/12	68/66	3:8/3:9	S	100 mg HSS 2 h prior surgery, 2 more doses given 8 h	PECA till day 2, acetaminophen and oral narcotic analgesics as needed	Adverse effects	hospital stay
Backes et al. [35]	2013	America	28/20	NR	NR	G	10 mg DXM prior to induction	Oral extended release oxycodone, celecoxib prior surgery, intraarticular pain pumps of 0.5% bupivacaine, PCA hydromorphone, oral extended release oxycodone, hydrocodone/acetaminophen and/or IV hydromorphone as request.	ROM	48 h
Koh et al. [36]	2013	Korea	135/134	72.0/72.0	38:117/35:119	S	10 mg DXM 1 h prior surgery	MOAG prior surgery (sustained-release oxycodone, celecoxib, pregabalin and acetaminophen), Continuous FNB, PIA, PCA fentanyl, Oral celecoxib, pregabalin and acetaminophen or IM of ketoprofen as required.	VAS and adverse effects	1 year
Lunn et al. [37]	2011	Denmark	24/24	66/67	13:11/8:16	S	125 mg MP prior anesthesia	LIA, IV sufentanil in PACU, oral oxycodone, celecoxib and slow-release acetaminophen, oral gabapentin and from the night of surgery up to the 6th.	VAS, Inflammatory cytokines and adverse effects	30 days
Jules-Elysee et al. [38]	2011	America	15/15^c^	64 /71	6:9/9:6	S	100 mg HC prior surgery, 2 dose eight hours apart	PECA(bupivacaine and hydromorphone), bilateral FNBs, oxycodone and acetaminophen as needed	VAS, Inflammatory cytokines and adverse effects	6 months
Peng et al. [39]	2021	China	60/60^c^	65.1/65.1	4:56/4:56	NR	7 mg betamethasone	PIA, PCA (morphine), parecoxib after for 3 days and oral Loxoprofen Sodium, aminophenol oxycodone sed as emergency.	VAS, ROM and adverse effects	3 months
Wang et al. [40]	2021	China	52/50	65.1/63.9	17:35/15:35	G	10 mg DXM	LIA, celecoxib, morphine hydrochloride injected subcutaneously as required.	VAS, ROM, Inflammatory cytokines and adverse effects	3 months
El-Boghdadly et al. [41]	2021	United Kingdom	68/72^a^	63.1/65.8	34:34/25:47	S	8 mg DXM	Oral acetaminophen and celecoxib preoperatively, LIA, IV fentanyl or hydromorphone at PACU, oral acetaminophen celecoxib. Oral oxycodone, hydromorphone or PCA with hydromorphone or morphine as request.	VAS, ROM and adverse effects	1 month
Chan et al. [42]	2020	China	45/45^c^	66/66	31:14:/31:14	S	40 mg TA	LIA, PCA.	VAS, ROM and adverse effects	1 year
Tsukada et al. [43]	2016	Japan	40/37	75 /72	5:35/5:32	S	40 mg MP	LIA, IV flurbiprofen axetil, oral loxoprofen, diclofenac sodium as request.	VAS, ROM and adverse effects	1 year
Kim et al. [44]	2015	Korea	43/43	71.4/70.6	2:41/4:39	S	40 mg MP	Oral celecoxib and tramadol/acetaminophen for pre 1 day, LIA, PCA (fentanyl), Ketorolac and fentanyl patch for 3—6 day. Oral celecoxib, tramadol/acetaminophen. IM of pethidine or oral oxycodone was used on rescue.	VAS, ROM, Inflammatory cytokines and adverse effects	7 days
Ikeuchi et al. [45]	2014	Japan	20/20	77 /76	2:18/4:16	G	6.6 mg DXM	LIA, PCA (Fentanyl), oral loxoprofen tablets until POD5 and as needed.	VAS, Inflammatory cytokines and adverse effects	3 months
Kwon et al. [46]	2014	Korea	76/76^c^	69.3/69.3	0:76/0:76	S	40 mg TA	Oral celecoxib, ultracet and pregabalin prior operation, LIA, PCA (fentanyl and bupivacaine), oral celecoxib and ultracet bid. IM of ketoprofen as request.	Adverse effects	6 months
Yue et al. [47]	2013	China	36/36	70.2/69.3	4:32/4:32	G	1 ml betamethasone	Oral celecoxib preoperatively and postoperatively, LIA, PCA (morphine), IM of morphine as required.	VAS, ROM and adverse effects	1 year
Chia et al. [48]	2013	Australia	39/40	68.9 /65.1	NR	S	40 mg triamcinolone acetate	LIA, celecoxib for 2 weeks.	VAS, ROM and adverse effects	3 months
Seah et al. [49]	2011	Singapore	50/50	67.9/65.4	NR	S/G	40 mg TA	LIA, oral naproxen and PCA (morphine).	VAS, ROM and adverse effects	2 year
Christensen et al. [50]	2009	America	39/37	65.8/65.2	16:23/7:30	G	40 mg MP acetate	Oral celecoxib, oxycodone and acetaminophen preoperatively, FNB, LIA,	VAS, ROM and adverse effects	3 months
Li et al. [51]	2021	China	45/45	66.1/68.2	15:30/8:37	G	10 mg DXM at the induction, 10 mg DXM, 100 ml	Oral loxoprofen or celecoxib after hospital admission, LIA, oral Loxoprofen, IM parecoxib until hospital discharge, IM Morphine hydrochloride as required.	VAS, ROM, Inflammatory cytokines and adverse effects	3 months
Hatayama et al. [52]	2021	Japan	50/50	71.6 /72.3	12:38/5:45	G	10 mg DXM 1 h prior, 24 h after surgery, 40 mg TA	PIA, FNB, oral acetaminophen on pod1, IM pentazocine and transrectal administration of diclofenac as request.	VAS, Inflammatory cytokines and adverse effects	1 week

*I/C* Interventional group/Control group, *M/F* Male/Female, *NR* not rated, *S/G* Intraspinal anesthesia/General anesthesia, *DXM* dexamethasone, *MP* methylprednisolone, *HC* hydrocortisone, *TA* triamcinolone acetonide, *HSS* hydrocortisone sodium succinate, *LIA* local infiltration analgesia, PIA periarticular infiltration analgesia, *PCA* patient-controlled intravenous analgesia, *PCEA* patient-controlled epidural analgesia, *MOAG* multimodal oral analgesic drugs, *IM* muscular injection, *IV* intravenous injection; FNB, femoral nerve block, *VAS* Visual analgesia score, *ROM* Range of Motion

^a^Final analyzed

^b^Knee replacement surgery

^c^Bilateral TKA

### Postoperative resting VAS

**Fig. 3:**
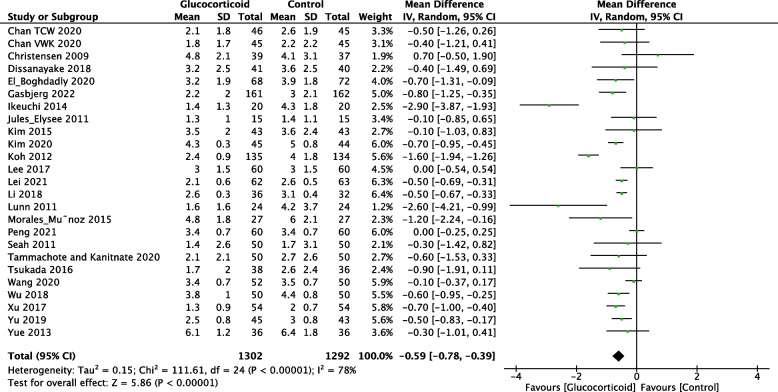
Forest plot of the effect of glucocorticoid on VAS score at rest on POD1 after TKA

**Fig. 4:**
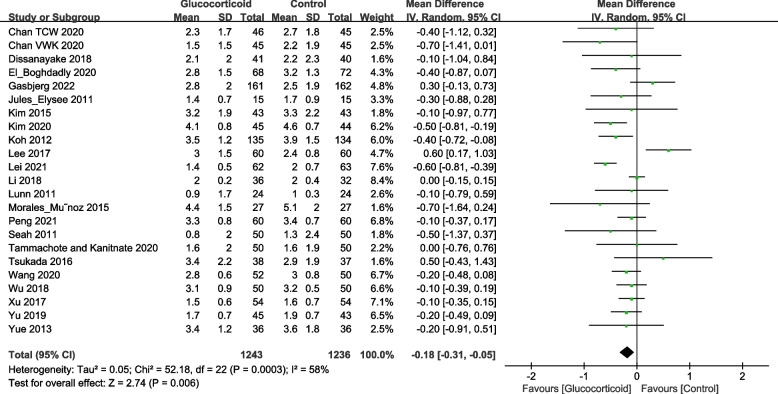
Forest plot of the effect of glucocorticoid on VAS score at rest on POD2 after TKA

**Fig. 5:**
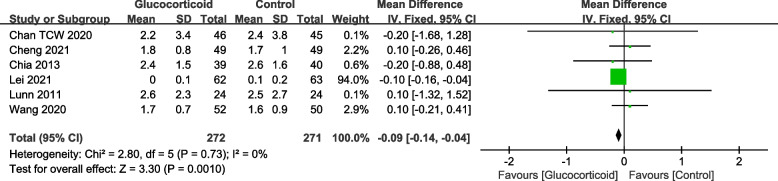
Forest plot of the effect of glucocorticoid on VAS score at rest on POM3 after TKA

The Visual Analogue Scale (VAS) is a tool commonly used to assess pain scales. It is an approximately 10 cm long ruler, divided into 10 equal scores ranging from 0 (pain-free end) to 10 (most severe pain). The patient select the appropriate scale on the ruler to indicate their level of pain. In the analysis, twenty-five studies reported the resting VAS on postoperative day 1 (POD1) following TKA, including 1,302 patients in the glucocorticoid group and 1,292 patients in the control group. Furthermore, twenty-three trials recorded the resting VAS on postoperative day 2 (POD2), with 1,243 patients in the glucocorticoid group and 1,236 patients in the control group. Six studies measured the resting VAS score on postoperative 3 months (POM3), including 272 patients in the glucocorticoid group and 271 patients in the control group. The results indicated that the use of glucocorticoids significantly reduced the postoperative resting VAS (Fig. 3, POD1: MD = -0.59; 95%CI: -0.78, -0.39; *P* < 0.000; Fig. 4, POD2: MD = -0.18; 95%CI: -0.31, -0.05;* P* = 0.006; Fig. 5, POM3: MD = -0.09; 95%CI: -0.14, -0.04; *P* = 0.001).

### Morphine consumption

**Fig. 6:**
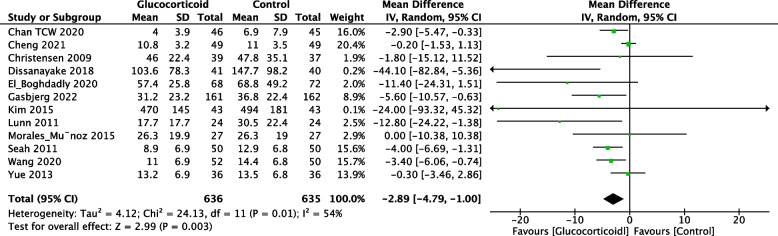
Forest plot of the effect of glucocorticoid on morphine consumption (mg) within 24 h after TKA

Eleven records provided data of morphine consumption within 24 h following TKA. The unit of morphine is milligram (mg). These studies involved 636 patients in the glucocorticoid group and 635 patients in the control group. Morphine consumption was significantly reduced in the glucocorticoid group (Fig. 6, MD = -2.89; 95% CI: -4.79 -1.00; *P* = 0.003) compared to the control group.

### Postoperative Range of Motion

**Fig. 7:**
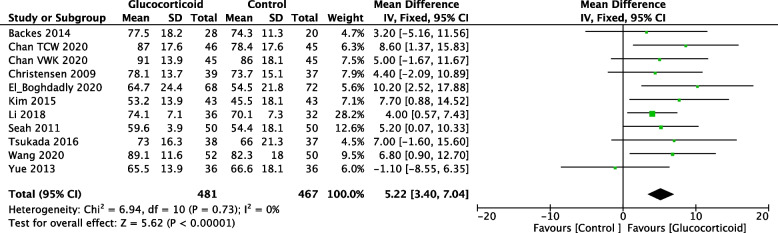
Forest plot of the effect of glucocorticoid on ROM (degrees) on POD1 after TKA

**Fig. 8:**
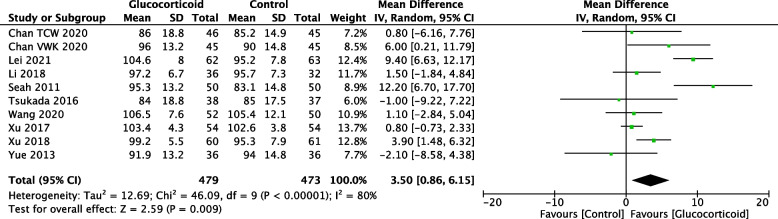
Forest plot of the effect of glucocorticoid on ROM (degrees) on POD3 after TKA

ROM refers to the range of motion of the knee in flexion, which is normally 0–130 angles. ROM in the knee joint was assessed on POD1 and postoperative days 3 (POD3). Eleven studies provided ROM data on POD1 with 481 patients in the glucocorticoid group and 467 patients in the control group. Ten studies assessed ROM on POD3, with 479 patients in the glucocorticoid group and 473 patients in the control group. The overall analysis demonstrated that systemic administration (SA) and periarticular injection analgesia (PIA) of glucocorticoids improved the ROM comparing to the control group after TKA (Fig. 7, POD1: MD = 5.22; 95%CI: 3.40, 7.04; *P* < 0.000; Fig. 8, POD3: MD = 3.50; 95%CI: 0.86, 6.15; *P* = 0.009).

### Postoperative inflammatory response

Eleven trials evaluated the C-reactive protein (CRP mmol/L) concentration on POD1, involving 487 patients in the glucocorticoid group and 482 patients in control group. Additionally, eleven trials recorded the CRP concentration on POD2, with 526 patients *vs.* 521 patients in two groups respectively. Seven studies reported the CRP concentration on POD3, encompassing 322 patients in the glucocorticoid group and 323 patients in the control group. The results showed a significant reduction of CRP in the glucocorticoid group compared to the control group (Supplementary Fig. 1, POD1: MD = -18.75; 95% CI: -23.36, -14.15; *P* < 0.000; Supplementary Fig. 2, POD2: MD: -47.05; 95% CI: -59.80, -34.29;* P* < 0.000; Supplementary Fig. 3, POD3: MD = -37.79; 95% CI: -49.45, -26.12; *P* < 0.000).

In addition, eight trials assessed the Interleukin-6 (IL-6 mmol/L) concentration on POD1, with 369 patients in the glucocorticoid group and 368 patients in the control group. The IL-6 concentration was significantly lower in the glucocorticoid group on POD1 (Supplementary Fig. 4, MD = -60.02; 95% CI: -71.02, -49.02; *P* < 0.000).

### Postoperative adverse effects

Data from seven studies were analyzed to investigate the incidence of postoperative nausea and vomiting (PONV) on POD1, while thirteen studies reported the PONV incidence during the entire postoperative period (from post-surgery to discharge). The results showed that perioperative systemic or periarticular injection glucocorticoid reduced the incidence of PONV not only on POD1 but also during total postoperative period (Supplementary Fig. 5, POD1: RR = 0.51; 95% CI: 0.43, 0.62; *P* < 0.000; Supplementary Fig. 6, Total: RR = 0.64; 95% CI: 0.51, 0.81; *P* = 0.0002).

In order to investigate the impact of glucocorticoid dosage on PONV, we conducted a reanalysis of the relevant data after applying dexamethasone equivalents. The results revealed that the majority of studies examining total PONV predominantly utilized low-dose glucocorticoid with dexamethasone equivalents maximum limit of 15 mg during entire perioperative period, 3 studies utilized high-dose dexamethasone equivalents. Both high-dose and low-dose glucocorticoid therapy can improve the incidence of total PONV (Supplementary Fig. 7, high-dose: RR = 0.42, *P* < 0.000; low-dose: RR = 0.72, *P* = 0.006), with a significant lower incidence of PONV in the high-dose group (Supplementary Fig. 7, *P* = 0.03).

Eight records provided data on blood glucose concentration on POD1, and five of them reported the blood glucose concentration on POD2 as well. The analysis showed that blood glucose concentration (mmol/L) was significantly increased in the glucocorticoid group on POD1 compared with the control group (Supplementary Fig. 8, MD = 0.47; 95%CI: 0.25, 0.68; *P* < 0.000). However, the difference in blood glucose concentration (mmol/L) between the two groups was reduced on POD2 (Supplementary Fig. 9, MD = -0.16; 95%CI: -0.55, 0.23; *P* = 0.42).

Fourteen studies evaluated the occurrence of wound infection postoperatively, while two studies reported the occurrence of venous thrombosis. The analysis did not show a significant increase in the risk of postoperative wound infection (Supplementary Fig. 10, RR = 0.98; 95% CI: 0.64, 1.51; *P* = 0.93) and venous thrombosis in the glucocorticoid group (Supplementary Fig. 11, RR = 1.47; 95% CI: 0.25, 8.68; *P* = 0.67).

### Length of hospital stay

Nineteen trials reported the duration of hospitalization after TKA which including 807 patients in the glucocorticoid group and 801 patients in the control group. The overall pooled outcomes showed that perioperative glucocorticoid administration significantly decreased the days of LOS comparing to the control group (Supplementary Fig. 12, MD = -0.27; 95% CI: -0.44, -0.09; *P* = 0.003).

### Comparison of efficacy between systemic administration and periarticular infiltration in glucocorticoid group

In the comparison between systemic administration (SA) and periarticular infiltration analgesia (PIA) of glucocorticoids, two studies with 95 patients in each group were included. The results showed that the postoperative resting VAS was significantly lower in periarticular infiltration group compare to the systemic administration group on POD2 (Supplementary Fig. 13, MD = -0.16; 95% CI: -0.29, -0.02; *P* = 0.02). However, the plasma CRP level was significantly lower in the systemic administration group compare to the periarticular infiltration group on POD1 and POD2 (Supplementary Fig. 14, POD1: MD = 10.30; 95% CI: 4.74, 15.85; *P* = 0.0003; Supplementary Fig. 15, POD2: MD = 13.62; 95% CI: 2.19, 25.05; *P* = 0.02).

In order to better explore the effects of different glucocorticoids administration routes, apart from the two aforementioned RCTs directly comparing the postoperative pain and peripheral blood inflammatory factors, we conducted subgroup analysis between the SA group and PIA group. Comparing to the control group, both subgroups showed a significant reduction in pain scores on POD1 without Inter-group difference (Supplementary Fig. 16–1, *P* = 0.38).The results also revealed that the PIA group exhibited a significant alleviation of postoperative resting VAS on POD2 (Supplementary Fig. 16–2, MD = -0.2; 95% CI: -0.36, -0.04; *P* = 0.01). Meanwhile, there was no statistically significant difference in the SA group on POD2. Totally, the reduction of resting VAS on POD2 was without Inter-group difference between SA and PIA group (Supplementary Fig. 16–2, *P* = 0.77). The comparison of inflammation cytokines levels between subgroups did not yield accurate results, primarily due to the substantial difference in sample sizes between the two subgroups.

### Publication bias

Publication bias was evaluated using funnel plots and Begg’s and Egger’s tests for VAS on POD1, 2, 3, which showed no evidence of publication bias (Supplementary Figs. 17, 18 and 19, Begg: Pr >|z|= 0.624, 0.635, 0.976, Egger’s: *P* = 0.491, 0.251, 0.733). And the publication bias for VAS on POM3 and morphine consumption were not assessed due to the small number of included studies.

## Discussion

The most important finding of the study is that the glucocorticoids can significantly reduce acute pain and morphine consumption within 24 h, and decrease the postoperative level of CRP and IL-6 and the incidence of PONV, and achieve a better ROM and shorter LOS, without increasing continued high blood glucose and the risk of postoperative wound infection and venous thrombosis.

Many patients experience severe pain after TKA surgery, and a significant number continue to suffer from chronic pain even after 3 months [53, 54]. To address this issue, numerous studies have investigated various postoperative analgesia protocols, such as the use of gabapentin [55] or dexmedetomidine [56] as adjuncts, as well as different combinations of nerve blocks [57]. Glucocorticoids have gained increasing attention as adjuvants for acute postoperative pain treatment in TKA [58]. In the current study, both intravenous and periarticular injection of glucocorticoids resulted in a significant decrease of 0.59 mean difference (MD) in resting pain scores on POD1 after surgery, which was consistent with previous studies [59–62]. While the MD of improvement of the resting VAS on POM3 was 0.09. However, similar MD in improvement of resting VAS scores showed no statistically significant difference in other studies [61, 62]. Therefore the effect of glucocorticoids on chronic pain need to be further explored. Morphine consumption was also reduced in the glucocorticoid group on POD1 which was similar to the morphine reduction of 3.4 mg in Wang et al. study [61]. These findings are consistent with a meta-analysis conducted by De Oliveira et al., which concluded that intermediate and high doses of perioperative dexamethasone can effectively reduce postoperative pain and the need for additional painkillers [63]. They also found that preoperative dexamethasone was more effective in pain control compared to intraoperative administration. Similarly, Waldron et al. demonstrated the pain-relieving effect of perioperative dexamethasone in various surgical procedures, showing that patients who received dexamethasone required fewer postoperative opioids, had a longer time to first analgesic dose, needed less rescue analgesia, and had shorter stays in the recovery room [64]. In conclusion, glucocorticoid applications are effective for short-term pain control but long-term control needs to be explored further.

In this study, the beneficial effects of glucocorticoid administration extended beyond pain relief. It was observed that perioperative glucocorticoids administration significantly improved ROM and decreased the LOS, which is consistent with previous studies [65]. Patients with total knee osteoarthritis often have decreased ROM due to joint stiffness and pain. ROM is a significant parameter evaluating postoperative functional recovery for patients after TKA [66]. Better ROM is more benefit to different activities of daily living [67]and has a positive impact on TKA outcomes [68]. In our study, the glucocorticoid group presented about 5.22 degrees on POD1 and 3.50 degrees on POD3 increased in the ROM after TKA. Though these results were also confirmed in some studies [40, 59], a study from Xu B et indicated that the ROM on POD3 was not significantly increasing after applying glucocorticoids [30]. Glucocorticoid was found no significant improvement in long-term keen function of TKA patients due to it short-lasting pain relief [59]. In general, Glucocorticoids could improve short-term ROM contributing to better functional recovery [69]. Because many other variables may influence postoperative ROM, including surgical technique, implant design, and preoperative training as well as postoperative care, further investigation is required, including follow up for long-term ROM.

LOS is deemed as an important outcome for its economics burden [70]. Our study has showed that glucocorticoids shorten postoperative LOS by 0.27 day, which is lessen than 1.2 days in the study from Backes et al. [35] LOS is affected by many factors, including Enhanced Recovery After Surgery (ERAS), persistent postoperative pain, nausea and vomiting, wound infection, as well as the administration times and dose of glucocorticoids. As studies attention intensifies on ERAS, encompassing routine education, smoking and alcohol cessation, optimized fasting periods, standardized multimodal anesthesia protocols, alongside the utilization of local infiltrative analgesia, tranexamic acid, and multimodal prophylactic treatment for postoperative nausea, vomiting, and analgesia, a notable reduction in LOS by 5.4 days following TKA is achievable [71]. Therefore, further studies are warranted to substantiate the efficacy of combining glucocorticoids with ERAS in reducing Length of Stay.

Our study also found that periarticular injection analgesia group had lower pain score on POD2 but higher CRP level on POD 1 and POD2 compared to the systemic administration group in the two comparative studies. And in a subgroup analysis of the route of glucocorticoid administration across included studies, PIA group indicated decreased resting VAS on POD2, while the SA group didn’t. Previous studies have used glucocorticoids via different routes, with intravenous or topical administration being commonly used and proven to accelerate recovery after TKA [72, 73]. The different effects of intravenous or topical glucocorticoids in TKA patients may be explained by the analgesic mechanism of glucocorticoids. Glucocorticoids exert their analgesic effect by inhibiting phospholipase, which blocks the cyclooxygenase and lipoxygenase pathways in the inflammatory chain reaction [74–76]. This leads to a decrease in the release of pain-causing substances such as bradykinin [74] from tissues and neuropeptides from nerve endings [75]. From this perspective, topical glucocorticoids directly inhibit the production of inflammatory factors, resulting in a faster and more targeted reduction of pain-causing substances such as bradykinin, compared to intravenous glucocorticoids [45, 51]. However, the advantages of locally administered glucocorticoids following TKA have not been conclusively demonstrated. Some studies have indicated that the use of local glucocorticoids results in prolonged pain relief and improved active knee flexion after surgery [77–79]. On the other hand, a study by Christensen et al. [50] did not observe improvements on pain scores, range of knee motion, or narcotic consumption with local glucocorticoid administration. As similar in our study, there were insignificant difference of the resting VAS on POD1 and POD2 between PIA and SA group across all studies. These conflicting results may be attributed to differences in disease severity among the study groups, variations in treatments prior to TKA, differences in the type of steroid used, concomitant analgesic drugs, and variations in postoperative physical therapy approaches, which can influence the range of motion outcomes.

Indeed, TKA is associated with a profound inflammatory response due to the deep surgical site, osteotomy, massive blood loss, soft tissue injury, and surgical-related stress response. This led to increased levels of various inflammatory cytokines including CRP, IL-1β, IL-6, and TNF-a [80, 81]. The systemic and local inflammatory responses contribute to hyperalgesia [82, 83] and pain sensitization after TKA, which can result in severe pain even with a slight stimuli [30, 84]. Furthermore, the persistent inflammatory response can contribute to the development of chronic pain in the postoperative period [85]. Glucocorticoid had been wildly used in TKA to enhance recovery due to their potent anti-inflammatory effects [30, 72, 86]. The use of glucocorticoids can reduce CRP and IL6 level in the blood, as observed in our study. The analgesic effects of preoperative glucocorticoids can be partly attributed to their reduction of inflammation [87]. Glucocorticoids exert their anti-inflammatory response by suppressing the transcription of AP-1, NF-kB, and STAT3, which leads to the inhibition of proinflammatory cytokine production [88]. However, topical administration of glucocorticoid group showed higher CRP level postoperatively compared to system administration of glucocorticoid group in our study. The administration route we chose were closely associated with local or systemic inflammatory response. Intravenous corticosteroids have been proved to reduce systemic CRP and IL-6 effectively [30, 72, 86], while topical corticosteroids predominantly improve local inflammation at the surgical site in arthroplasty [45] and have limited effects on system inflammation after TKA [73]. However, these findings need to be confirmed through additional clinical studies, as there are only two comparative studies presented in this paper. Study by Ugras et al. proposed that amelioration of local inflammation is more significant in the analgesia of glucocorticoid than system inflammation [80], which is consistent with our results that topical administration of glucocorticoid has lower pain score on POD2. However, more studies are required to support it.

PONV commonly occur in TKA patients and can hinder participation in physiotherapy and early mobilization [89], leading to prolonged hospital stays and increasing the dissatisfaction of patients and health care costs [90, 91]. Glucocorticoids have been utilized in the prevention of PONV in many studies [92]. We found that the glucocorticoids have anti-emetic function after TKA not only in system administration but in local usage. The anti-emetic effect of glucocorticoids may be mediated through the inhibition of prostaglandin synthesis or the inhibition of endogenous opioid release [93]. Meanwhile, surgical trauma, anesthesia, and analgesics [94] are all factors associated with PONV, and surgical trauma can trigger the release of systemic inflammatory biomarkers, thereby increasing the occurrence of PONV [95]. The potent anti-inflammatory effects of glucocorticoids may contribute to their anti-emetic function. Though the anti-emetic of glucocorticoid has been well documented and supported in our study, the application dosage is still not clarified clearly. Therefore, we performed subgroup analysis of glucocorticoid dose and concluded that low dosage (≤ 15 mg dexamethasone) and high dosage (> 15 mg dexamethasone) both reduced the incidence of PONV, with high dosage reducing PONV more significantly. And pre-emptive low dose (10 mg) dexamethasone has been proved to reduce postoperative emesis [36]. Though higher dosage of glucocorticoid is more beneficial in reducing PONV, we cannot ignore higher risk of complications with it. It has so far been proposed multiple low dosage of glucocorticoid is more effective on decrease PONV and early rehabilitation [59, 86]. However, a single high dose of 20 mg dexamethasone is more preferable to multiple low dose application by an opposite study [96]. It may be related to the type of glucocorticoid, as they have different times of action and should therefore be used specifically according to the type of glucocorticoid in clinical.

In addition, referring to the concern of hyperglycemia caused by the administration of corticosteroids, previous studies indicated that the fasting blood glucose of patients treated with topical and intravenous glucocorticoid was significantly increased after TKA [52, 97, 98], which was similar to significant elevation of blood glucose in the glucocorticoid group on POD1 in the study. Hyperglycemia, especially when levels exceed 200 mg/dl has been associated with an increased risk of surgical site infection and wound complications [99, 100]. However, other studies reported that the intravenous administration of dexamethasone did not increase fasting blood glucose levels postoperatively following TKA [35, 101], which were consistent with the insignificant increase blood glucose in patients of glucocorticoid group after TKA on POD2. Different results may be related to the determination time of blood glucose and Hormone dosage. It also indicated that preoperative intravenous administration of methylprednisolone resulted in a transient postoperative increase in plasma glucose and insulin resistance, which were normalized 48 h postoperatively [97].

The other complications in terms of wound drainage, delayed wound healing, intramuscular venous thrombosis, which were not significantly different between the two groups. To our knowledge, most important possible risks with steroid during post-operative period, including gastric ulcers [102], impaired wound healing [103] or wound infections [11] were exclusively associated with chronic glucocorticoid use not with single dose glucocorticoid use. And the side effects from glucocorticoid use are proportional to the duration and intensity of therapy [104]. Theses similar results were presented in many articles [105]. Even though, no studies have confirmed that a short term use of glucocorticoid has not harm effect on patients of TKA. In addition, it may be small samples and low evidenced articles that result in negative adverse outcomes in single dose of glucocorticoid use [106]. Therefore, further high-quality studies with larger sample sizes is needed to establish the safety of perioperative glucocorticoid administration in TKA patients.

We have investigated the clinical efficacy of most glucocorticoid, but the optimal corticosteroids administration regimen regarding of dosage, times, drug combination are still undetermined. In concern of the comparison between topical and systemic glucocorticoid, there need more studies to explore their different effect on pain and system inflammation. Additionally, the lack of long-term follow-up data highlights the need for further studies to explore and validate the results over an extended period of time.

## Conclusion

This systematic review and meta-analysis suggest that glucocorticoids may exert pain relief within 24 h, although its clinical relevance to patients may be limited. The application of glucocorticoids provided antiemetic effects, as well as facilitate functional recovery, accompanied by the inhibition of inflammatory factors releasing after TKA, including plasma CRP and IL-6. Importantly, these effects are observed without elevating blood glucose levels persistently, increasing the risks of wound infection and venous thrombosis.

### Supplementary Information

**Supplementary Material 1.**

## Data Availability

All data generated or analyzed during this study are included in this published article.
