# Supplementary material for: The efficacy and safety of perioperative glucocorticoid for total knee arthroplasty: a systematic review and meta-analysis
Source: BMC Anesthesiol. 2024 Apr 15;24:144. doi: 10.1186/s12871-024-02530-9 (PMC11017604; doi:10.1186/s12871-024-02530-9)
Supplement: Supplementary file 1 — Supplementary Material 1. [file 12871_2024_2530_MOESM1_ESM.zip › Supplementary figures/Supplementary figure 16-2.pdf]

Study or Subgroup	Glucocorticoid Mean	SD	Total	Control Mean	SD	Total	Weight	Mean Difference IV, Random, 95% CI
**1.2.1 SA**								
Chan TCW 2020	2.3	1.7	46	2.7	1.8	45	2.5%	−0.40 [−1.12, 0.32]
Dissanayake 2018	2.1	2	41	2.2	2.3	40	1.6%	−0.10 [−1.04, 0.84]
Gasbjerg 2022	2.8	2	161	2.5	1.9	162	4.8%	0.30 [−0.13, 0.73]
Jules_Elysee 2011	1.4	0.7	15	1.7	0.9	15	3.3%	−0.30 [−0.88, 0.28]
Kim 2020	4.1	0.8	45	4.6	0.7	44	6.2%	−0.50 [−0.81, −0.19]
Koh 2012	3.5	1.2	135	3.9	1.5	134	6.0%	−0.40 [−0.72, −0.08]
Lee 2017	3	1.5	60	2.4	0.8	60	4.7%	0.60 [0.17, 1.03]
Lei 2021	1.4	0.5	62	2	0.7	63	7.6%	−0.60 [−0.81, −0.39]
Li 2018	2	0.2	36	2	0.4	32	8.4%	0.00 [−0.15, 0.15]
Lunn 2011	0.9	1.7	24	1	0.3	24	2.6%	−0.10 [−0.79, 0.59]
Morales_Mu˜noz 2015	4.4	1.5	27	5.1	2	27	1.6%	−0.70 [−1.64, 0.24]
Tammachote and Kanitnate 2020	1.6	2	50	1.6	1.9	50	2.2%	0.00 [−0.76, 0.76]
Wu 2018	3.1	0.9	50	3.2	0.5	50	6.6%	−0.10 [−0.39, 0.19]
Xu 2017	1.5	0.6	54	1.6	0.7	54	7.1%	−0.10 [−0.35, 0.15]
Yu 2019	1.7	0.7	45	1.9	0.7	43	6.5%	−0.20 [−0.49, 0.09]
**Subtotal (95% CI)**			**851**			**843**	**71.8%**	**−0.17 [−0.34, 0.01]**
Heterogeneity: $Tau^2 = 0.07$; $Chi^2 = 46.23$, df = 14 (P < 0.0001); $I^2 = 70\%$								
Test for overall effect: Z = 1.87 (P = 0.06)								
**1.2.2 PIA**								
Chan VWK 2020	1.5	1.5	45	2.2	1.9	45	2.5%	−0.70 [−1.41, 0.01]
El_Boghdadly 2020	2.8	1.5	68	3.2	1.3	72	4.3%	−0.40 [−0.87, 0.07]
Kim 2015	3.2	1.9	43	3.3	2.2	43	1.8%	−0.10 [−0.97, 0.77]
Peng 2021	3.3	0.8	60	3.4	0.7	60	6.8%	−0.10 [−0.37, 0.17]
Seah 2011	0.8	2	50	1.3	2.4	50	1.8%	−0.50 [−1.37, 0.37]
Tsukada 2016	3.4	2.2	38	2.9	1.9	37	1.6%	0.50 [−0.43, 1.43]
Wang 2020	2.8	0.6	52	3	0.8	50	6.7%	−0.20 [−0.48, 0.08]
Yue 2013	3.4	1.2	36	3.6	1.8	36	2.5%	−0.20 [−0.91, 0.51]
**Subtotal (95% CI)**			**392**			**393**	**28.2%**	**−0.20 [−0.36, −0.04]**
Heterogeneity: $Tau^2 = 0.00$; $Chi^2 = 5.85$, df = 7 (P = 0.56); $I^2 = 0\%$								
Test for overall effect: Z = 2.46 (P = 0.01)								
**Total (95% CI)**			**1243**			**1236**	**100.0%**	**−0.18 [−0.31, −0.05]**
Heterogeneity: $Tau^2 = 0.05$; $Chi^2 = 52.18$, df = 22 (P = 0.0003); $I^2 = 58\%$								
Test for overall effect: Z = 2.74 (P = 0.006)								
Test for subgroup differences: $Chi^2 = 0.08$, df = 1 (P = 0.77), $I^2 = 0\%$								

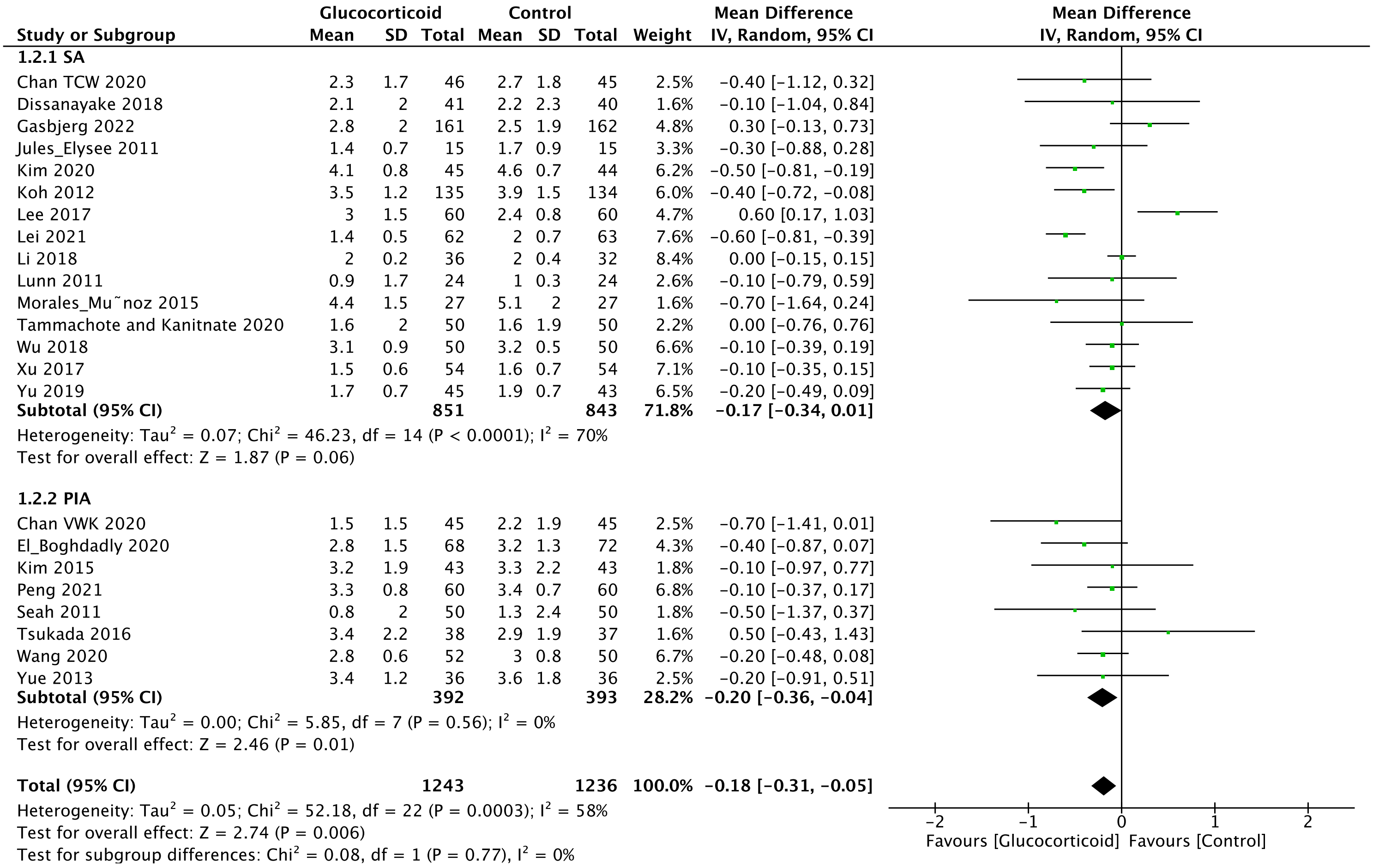

Figure S16-2. Forest plot of the effect of glucocorticoid on resting VAS on POD2 in SA and PIA group among included studies
