# Supplementary material for: The efficacy and safety of perioperative glucocorticoid for total knee arthroplasty: a systematic review and meta-analysis
Source: BMC Anesthesiol. 2024 Apr 15;24:144. doi: 10.1186/s12871-024-02530-9 (PMC11017604; doi:10.1186/s12871-024-02530-9)
Supplement: Supplementary file 1 — Supplementary Material 1. [file 12871_2024_2530_MOESM1_ESM.zip › Supplementary figures/Supplementary figure 18.pdf]

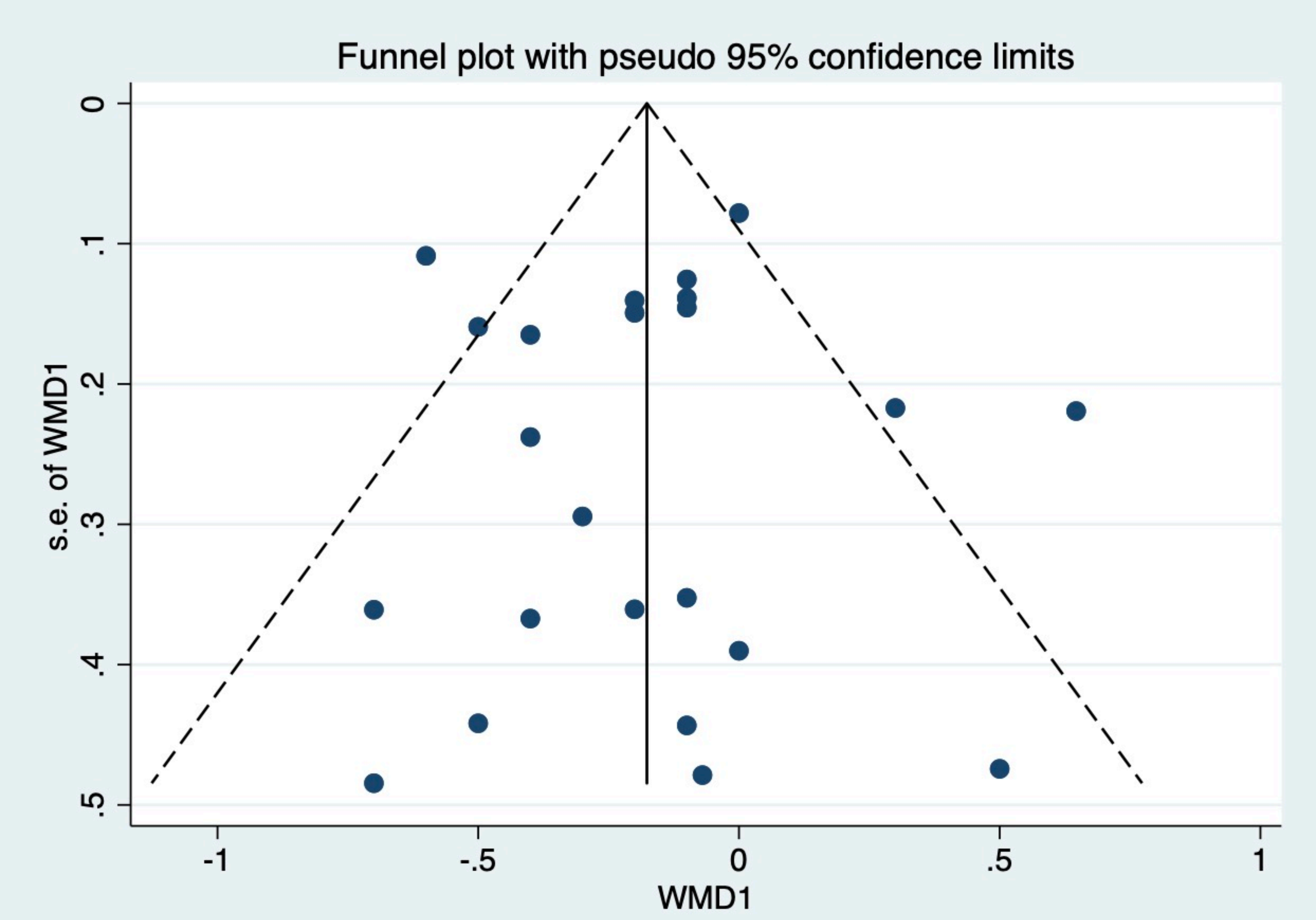

Figure S18. Funnel plot of the effect of glucocorticoid on resting VAS on POD2 after TKA
