# Supplementary material for: The efficacy and safety of perioperative glucocorticoid for total knee arthroplasty: a systematic review and meta-analysis
Source: BMC Anesthesiol. 2024 Apr 15;24:144. doi: 10.1186/s12871-024-02530-9 (PMC11017604; doi:10.1186/s12871-024-02530-9)
Supplement: Supplementary file 1 — Supplementary Material 1. [file 12871_2024_2530_MOESM1_ESM.zip › Supplementary figures/Supplementary figure 16-1.pdf]

Study or Subgroup	Glucocorticoid Mean	Glucocorticoid SD	Glucocorticoid Total	Control Mean	Control SD	Control Total	Weight	Mean Difference IV, Random, 95% CI
**1.1.1 SA**								
Chan TCW 2020	2.1	1.8	46	2.6	1.9	45	3.3%	−0.50 [−1.26, 0.26]
Dissanayake 2018	3.2	2.5	41	3.6	2.5	40	2.2%	−0.40 [−1.49, 0.69]
Gasbjerg 2022	2.2	2	161	3	2.1	162	5.0%	−0.80 [−1.25, −0.35]
Jules_Elysee 2011	1.3	1	15	1.4	1.1	15	3.4%	−0.10 [−0.85, 0.65]
Kim 2020	4.3	0.3	45	5	0.8	44	6.0%	−0.70 [−0.95, −0.45]
Koh 2012	2.4	0.9	135	4	1.8	134	5.6%	−1.60 [−1.94, −1.26]
Lee 2017	3	1.5	60	3	1.5	60	4.5%	0.00 [−0.54, 0.54]
Lei 2021	2.1	0.6	62	2.6	0.5	63	6.3%	−0.50 [−0.69, −0.31]
Li 2018	2.6	0.3	36	3.1	0.4	32	6.4%	−0.50 [−0.67, −0.33]
Lunn 2011	1.6	1.6	24	4.2	3.7	24	1.2%	−2.60 [−4.21, −0.99]
Morales_Mu˜noz 2015	4.8	1.8	27	6	2.1	27	2.3%	−1.20 [−2.24, −0.16]
Tammachote and Kanitnate 2020	2.1	2.1	50	2.7	2.6	50	2.7%	−0.60 [−1.53, 0.33]
Wu 2018	3.8	1	50	4.4	0.8	50	5.5%	−0.60 [−0.95, −0.25]
Xu 2017	1.3	0.9	54	2	0.7	54	5.8%	−0.70 [−1.00, −0.40]
Yu 2019	2.5	0.8	45	3	0.8	43	5.6%	−0.50 [−0.83, −0.17]
**Subtotal (95% CI)**			**851**			**843**	**65.6%**	**−0.67 [−0.87, −0.47]**
Heterogeneity: $Tau^2 = 0.09$; $Chi^2 = 51.18$, df = 14 (P < 0.00001); $I^2 = 73\%$								
Test for overall effect: Z = 6.50 (P < 0.00001)								
**1.1.2 PIA**								
Chan VWK 2020	1.8	1.7	45	2.2	2.2	45	3.1%	−0.40 [−1.21, 0.41]
Christensen 2009	4.8	2.1	39	4.1	3.1	37	1.9%	0.70 [−0.50, 1.90]
El_Boghdadly 2020	3.2	1.9	68	3.9	1.8	72	4.0%	−0.70 [−1.31, −0.09]
Ikeuchi 2014	1.4	1.3	20	4.3	1.8	20	2.5%	−2.90 [−3.87, −1.93]
Kim 2015	3.5	2	43	3.6	2.4	43	2.7%	−0.10 [−1.03, 0.83]
Peng 2021	3.4	0.7	60	3.4	0.7	60	6.0%	0.00 [−0.25, 0.25]
Seah 2011	1.4	2.6	50	1.7	3.1	50	2.1%	−0.30 [−1.42, 0.82]
Tsukada 2016	1.7	2	38	2.6	2.4	36	2.4%	−0.90 [−1.91, 0.11]
Wang 2020	3.4	0.7	52	3.5	0.7	50	5.9%	−0.10 [−0.37, 0.17]
Yue 2013	6.1	1.2	36	6.4	1.8	36	3.6%	−0.30 [−1.01, 0.41]
**Subtotal (95% CI)**			**451**			**449**	**34.4%**	**−0.46 [−0.87, −0.05]**
Heterogeneity: $Tau^2 = 0.27$; $Chi^2 = 39.49$, df = 9 (P < 0.00001); $I^2 = 77\%$								
Test for overall effect: Z = 2.22 (P = 0.03)								
**Total (95% CI)**			**1302**			**1292**	**100.0%**	**−0.59 [−0.78, −0.39]**
Heterogeneity: $Tau^2 = 0.15$; $Chi^2 = 111.61$, df = 24 (P < 0.00001); $I^2 = 78\%$								
Test for overall effect: Z = 5.86 (P < 0.00001)								
Test for subgroup differences: $Chi^2 = 0.78$, df = 1 (P = 0.38), $I^2 = 0\%$								

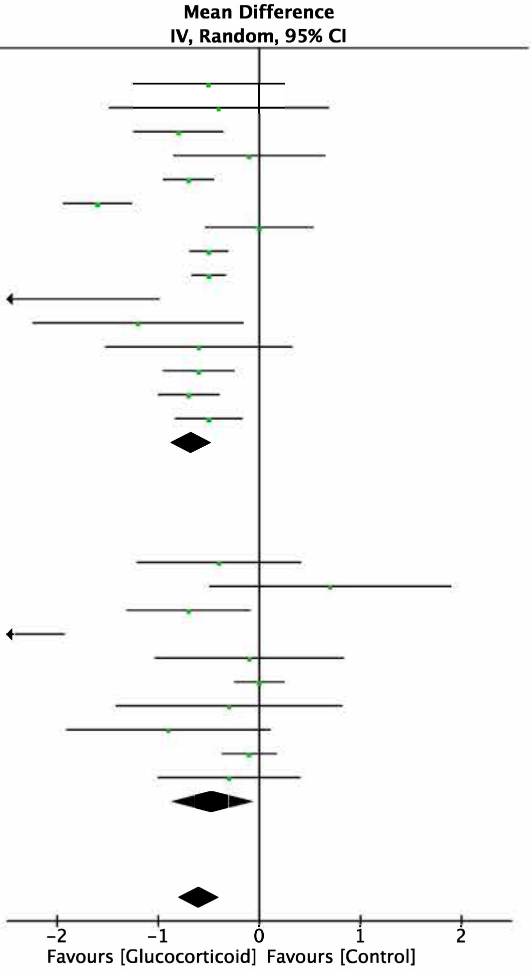

Figure S16-1. Forest plot of the effect of glucocorticoid on resting VAS on POD1 in SA and PIA group among included studies
