# Supplementary material for: The efficacy and safety of perioperative glucocorticoid for total knee arthroplasty: a systematic review and meta-analysis
Source: BMC Anesthesiol. 2024 Apr 15;24:144. doi: 10.1186/s12871-024-02530-9 (PMC11017604; doi:10.1186/s12871-024-02530-9)
Supplement: Supplementary file 1 — Supplementary Material 1. [file 12871_2024_2530_MOESM1_ESM.zip › Supplementary figures/Supplementary figure 6.pdf]

Study or Subgroup	Glucocorticoid Events	Glucocorticoid Total	Control Events	Control Total	Weight	Risk Ratio M-H, Random, 95% CI
Ikeuchi 2014	1	20	4	20	1.2%	0.25 [0.03, 2.05]
Jules_Elysee 2011	8	15	8	15	7.5%	1.00 [0.51, 1.95]
Kim 2015	24	43	21	42	12.4%	1.12 [0.75, 1.67]
Koh 2012	33	135	54	134	13.4%	0.61 [0.42, 0.87]
Kwon 2014	12	76	14	76	7.1%	0.86 [0.42, 1.73]
Lei 2021	2	62	11	63	2.2%	0.18 [0.04, 0.80]
Li 2018	13	36	15	32	9.0%	0.77 [0.44, 1.36]
Lunn 2011	8	24	15	24	7.8%	0.53 [0.28, 1.02]
Morales_Mu˜noz 2015	3	27	12	27	3.4%	0.25 [0.08, 0.79]
Tammachote and Kanitnate 2020	29	50	42	50	15.7%	0.69 [0.53, 0.90]
Wang 2020	14	52	18	50	8.9%	0.75 [0.42, 1.34]
Xu 2017	9	54	24	54	7.5%	0.38 [0.19, 0.73]
Xu 2018	4	60	14	61	3.9%	0.29 [0.10, 0.83]
**Total (95% CI)**		**654**		**648**	**100.0%**	**0.64 [0.51, 0.81]**
Total events	160		252			

Heterogeneity: $Tau^2 = 0.07$; $Chi^2 = 22.42$, df = 12 (P = 0.03); $I^2 = 46\%$

Test for overall effect: Z = 3.74 (P = 0.0002)

Risk Ratio
M-H, Random, 95% CI

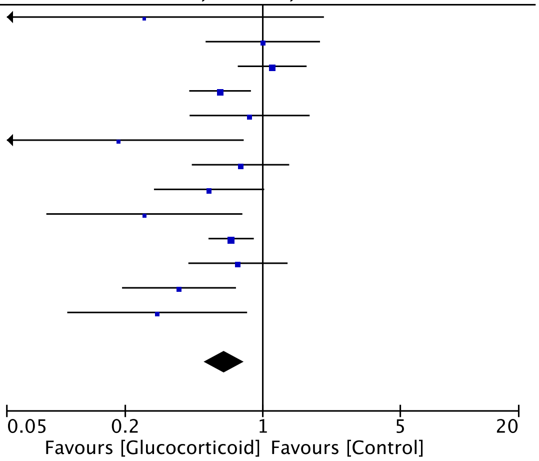

Figure S6. Forest plot of the effect of glucocorticoid on PONV during total postoperative period after TKA
