# Supplementary material for: The efficacy and safety of perioperative glucocorticoid for total knee arthroplasty: a systematic review and meta-analysis
Source: BMC Anesthesiol. 2024 Apr 15;24:144. doi: 10.1186/s12871-024-02530-9 (PMC11017604; doi:10.1186/s12871-024-02530-9)
Supplement: Supplementary file 1 — Supplementary Material 1. [file 12871_2024_2530_MOESM1_ESM.zip › Supplementary figures/SUPPLEMENTARY FIGURES LEGENDS.docx]

**SUPPLEMENTARY FIGURES LEGENDS**

Supplementary figure 1. Forest plot of the effect of glucocorticoid on plasma CRP (mmol/L) on POD1 after TKA

Supplementary figure 1. Forest plot of the effect of glucocorticoid on plasma CRP (mmol/L) on POD2 after TKA

Supplementary figure 1. Forest plot of the effect of glucocorticoid on plasma CRP (mmol/L) on POD3 after TKA

Supplementary figure 4. Forest plot of the effect of glucocorticoid on plasma IL-6 (mmol/L) on POD1 after TKA

Supplementary figure 5. Forest plot of the effect of glucocorticoid on PONV on POD1 after TKA

Supplementary figure 6. Forest plot of the effect of glucocorticoid on PONV during total postoperative period after TKA

Supplementary figure 7. Forest plot of the effect of different dosage of dexamethasone (mg) on PONV during total postoperative period after TKA

Supplementary figure 8. Forest plot of the effect of glucocorticoid on blood glucose (mmol/L) on POD1 after TKA

Supplementary figure 9. Forest plot of the effect of glucocorticoid on blood glucose (mmol/L) on POD2 after TKA

Supplementary figure 10. Forest plot of the effect of glucocorticoid on wound infection after TKA

Supplementary figure 11. Forest plot of the effect of glucocorticoid on venous thrombosis after TKA

Supplementary figure 12. Forest plot of the effect of glucocorticoid on LOS (days) after TKA

Supplementary figure 13. Forest plot of the effect of glucocorticoid on resting VAS on POD1 and POD2 between SA and PIA group in two comparative studies

Supplementary figure 14. Forest plot of the effect of glucocorticoid on plasma CRP (mmol/L) on POD1 between SA and PIA group in two comparative studies

Supplementary figure 15. Forest plot of the effect of glucocorticoid on plasma CRP (mmo/L) on POD2 SA and PIA group in two comparative studies

Supplementary figure 16-1. Forest plot of the effect of glucocorticoid on resting VAS on POD1 in SA and PIA group among included studies

Supplementary figure 16-2. Forest plot of the effect of glucocorticoid on resting VAS on POD2 in SA and PIA group among included studies

Supplementary figure 17. Funnel plot of the effect of glucocorticoid on resting VAS on POD1 after TKA

Supplementary figure 18. Funnel plot of the effect of glucocorticoid on resting VAS on POD2 after TKA

Supplementary figure 19. Funnel plot of the effect of glucocorticoid on resting VAS on POD3 after TKA
