# Supplementary material for: The efficacy and safety of perioperative glucocorticoid for total knee arthroplasty: a systematic review and meta-analysis
Source: BMC Anesthesiol. 2024 Apr 15;24:144. doi: 10.1186/s12871-024-02530-9 (PMC11017604; doi:10.1186/s12871-024-02530-9)
Supplement: Supplementary file 1 — Supplementary Material 1. [file 12871_2024_2530_MOESM1_ESM.zip › Supplementary figures/Supplementary figure 15.pdf]

Study or Subgroup	PIA Mean	PIA SD	PIA Total	SA Mean	SA SD	SA Total	Weight	Mean Difference IV, Fixed, 95% CI
Hatayama 2021	95	44	50	75	42	50	46.0%	20.00 [3.14, 36.86]
Li 2021	60.6	45	45	52.4	28.4	45	54.0%	8.20 [-7.35, 23.75]
**Total (95% CI)**			**95**			**95**	**100.0%**	**13.62 [2.19, 25.05]**

Heterogeneity: Chi² = 1.02, df = 1 (P = 0.31); I^2 = 2%
Test for overall effect: Z = 2.34 (P = 0.02)

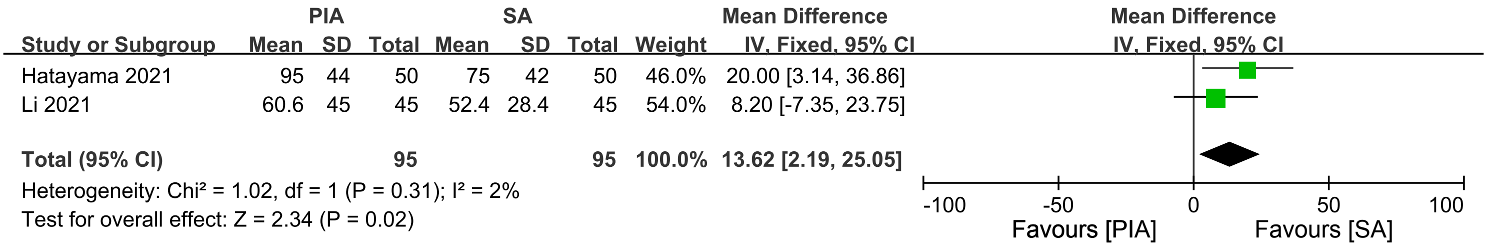

Figure S15. Forest plot of the effect of glucocorticoid on plasma CRP (mmo/L) on POD2 between SA and PIA group in two comparative studies
