# Supplementary material for: The efficacy and safety of perioperative glucocorticoid for total knee arthroplasty: a systematic review and meta-analysis
Source: BMC Anesthesiol. 2024 Apr 15;24:144. doi: 10.1186/s12871-024-02530-9 (PMC11017604; doi:10.1186/s12871-024-02530-9)
Supplement: Supplementary file 1 — Supplementary Material 1. [file 12871_2024_2530_MOESM1_ESM.zip › Supplementary figures/Supplementary figure 14.pdf]

Study or Subgroup	PIA Mean	PIA SD	PIA Total	SA Mean	SA SD	SA Total	Weight	Mean Difference IV, Fixed, 95% CI
Hatayama 2021	45	22	50	34	18	50	49.7%	11.00 [3.12, 18.88]
Li 2021	31.9	21.2	45	22.3	16.4	45	50.3%	9.60 [1.77, 17.43]
**Total (95% CI)**			**95**			**95**	**100.0%**	**10.30 [4.74, 15.85]**

Heterogeneity: Chi² = 0.06, df = 1 (P = 0.80); I² = 0%
Test for overall effect: Z = 3.63 (P = 0.0003)

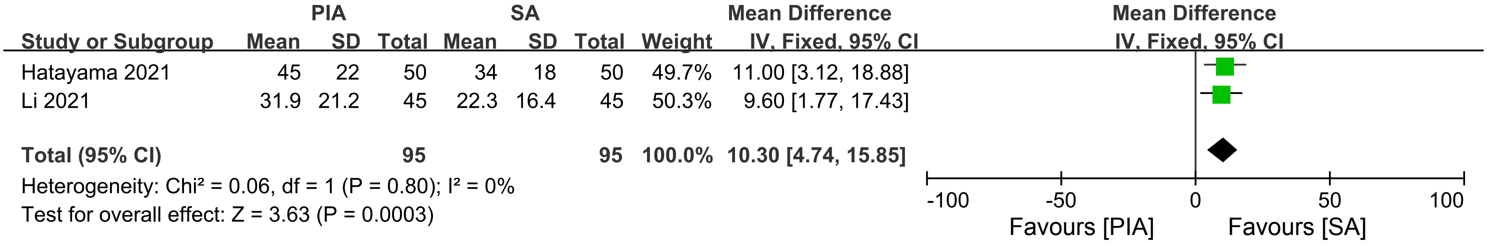

Figure S14. Forest plot of the effect of glucocorticoid on plasma CRP (mmol/L) on POD1 between SA and PIA group in two comparative studies
