# Supplementary material for: The efficacy and safety of perioperative glucocorticoid for total knee arthroplasty: a systematic review and meta-analysis
Source: BMC Anesthesiol. 2024 Apr 15;24:144. doi: 10.1186/s12871-024-02530-9 (PMC11017604; doi:10.1186/s12871-024-02530-9)
Supplement: Supplementary file 1 — Supplementary Material 1. [file 12871_2024_2530_MOESM1_ESM.zip › Supplementary figures/Supplementary figure 13.pdf]

Study or Subgroup	PIA Mean	PIA SD	PIA Total	SA Mean	SA SD	SA Total	Weight	Mean Difference IV, Random, 95% CI
Hatayama 2021	3.1	2	50	4.3	2.1	50	41.2%	−1.20 [−2.00, −0.40]
Li 2021	3	0.6	45	3.3	0.9	45	58.8%	−0.30 [−0.62, 0.02]
**Total (95% CI)**			**95**			**95**	**100.0%**	**−0.67 [−1.54, 0.20]**

Heterogeneity: $Tau^2 = 0.31$; $Chi^2 = 4.17$, df = 1 (P = 0.04); $I^2 = 76\%$
Test for overall effect: Z = 1.51 (P = 0.13)

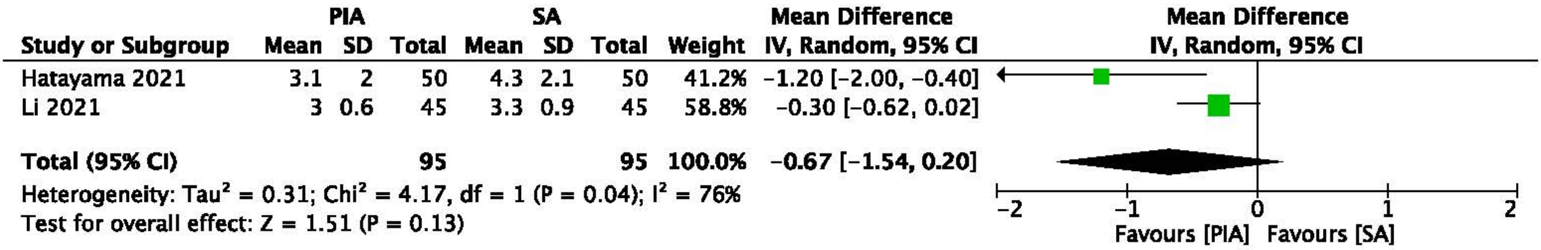

Study or Subgroup	PIA Mean	PIA SD	PIA Total	SA Mean	SA SD	SA Total	Weight	Mean Difference IV, Fixed, 95% CI
Hatayama 2021	2.2	0.4	50	2.4	0.5	50	57.5%	-0.20 [-0.38, -0.02]
Li 2021	2.4	0.5	45	2.5	0.5	45	42.5%	-0.10 [-0.31, 0.11]
**Total (95% CI)**			**95**			**95**	**100.0%**	**-0.16 [-0.29, -0.02]**

Heterogeneity: $Chi^2 = 0.52$, df = 1 (P = 0.47); $I^2 = 0\%$
Test for overall effect: Z = 2.29 (P = 0.02)

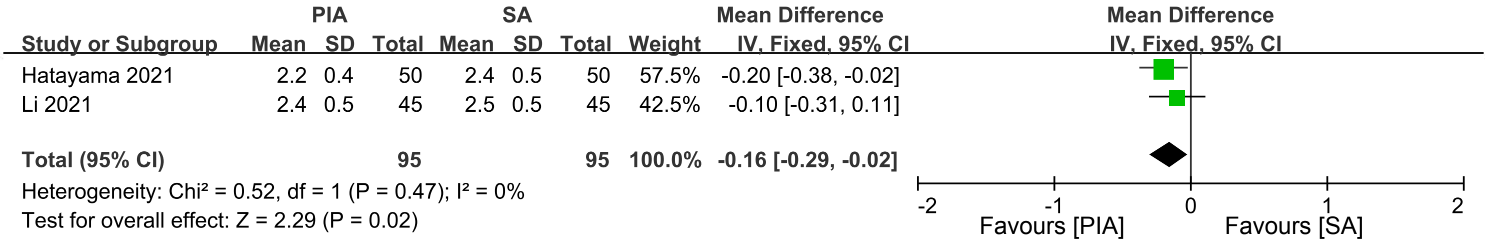

Figure S13. Forest plot of the effect of glucocorticoid on resting VAS on POD1 and POD2 between SA and PIA group in two comparative studies
