# Supplementary figures and images for: The efficacy and safety of perioperative glucocorticoid for total knee arthroplasty: a systematic review and meta-analysis
Source: BMC Anesthesiol. 2024 Apr 15;24:144. doi: 10.1186/s12871-024-02530-9 (PMC11017604; doi:10.1186/s12871-024-02530-9)

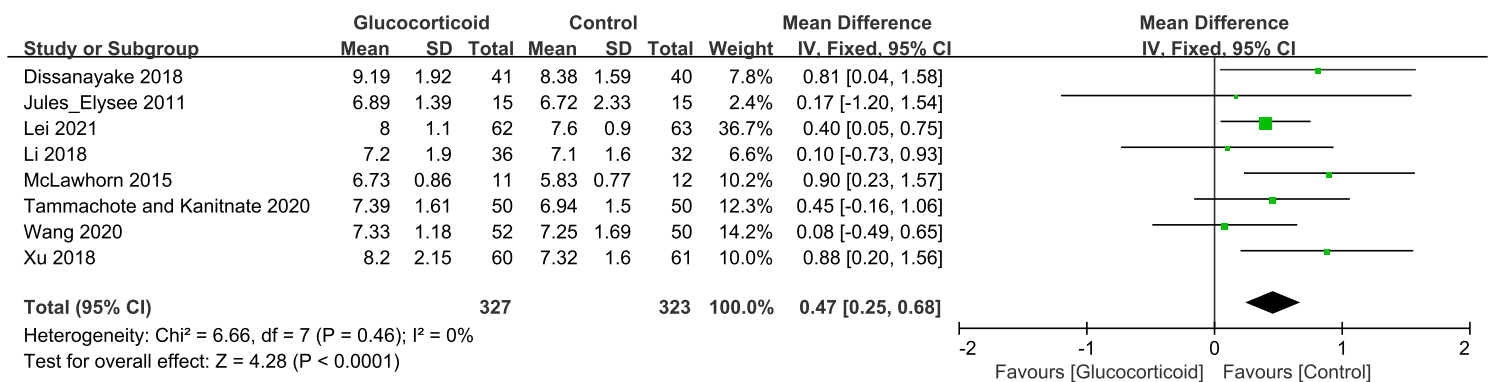

**Figure S8. Forest plot of the effect of glucocorticoid on blood glucose (mmol/L) on POD1 after TKA**

Supplement: Supplementary file 1 — Supplementary Material 1. [file 12871_2024_2530_MOESM1_ESM.zip › Supplementary figures/Supplementary figure 8.pdf]

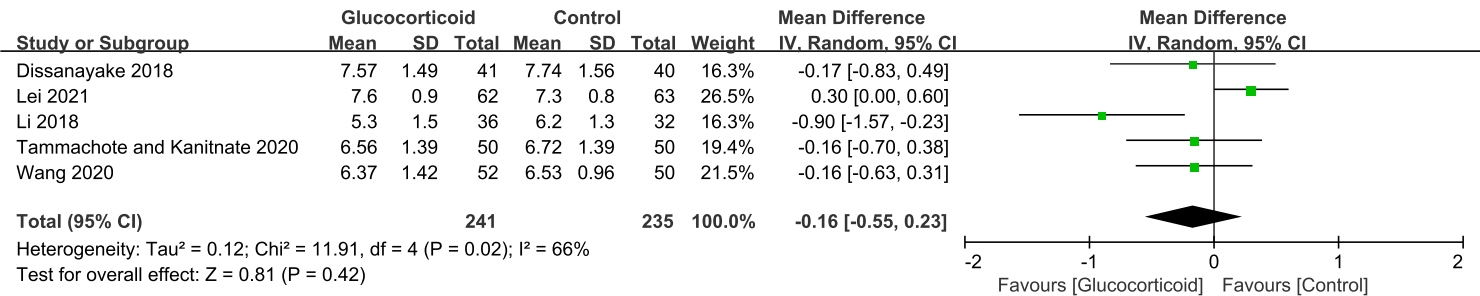

**Figure S9. Forest plot of the effect of glucocorticoid on blood glucose (mmol/L) on POD2 after TKA**

Supplement: Supplementary file 1 — Supplementary Material 1. [file 12871_2024_2530_MOESM1_ESM.zip › Supplementary figures/Supplementary figure 9.pdf]

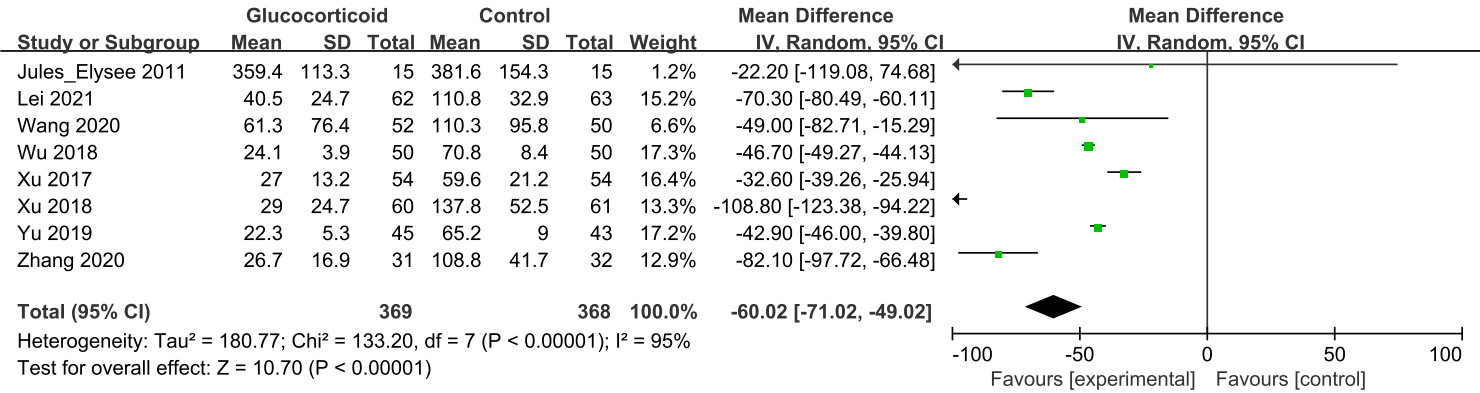

**Figure S4. Forest plot of the effect of glucocorticoid on plasma IL-6 (mmol/L) on POD1 after TKA**

Supplement: Supplementary file 1 — Supplementary Material 1. [file 12871_2024_2530_MOESM1_ESM.zip › Supplementary figures/Supplementary figure 4.pdf]

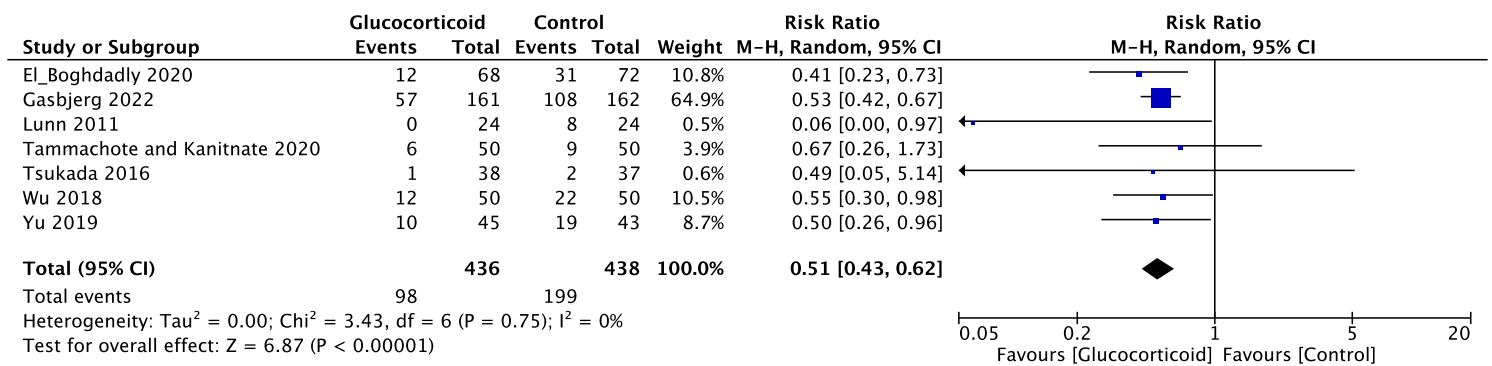

**Figure S5. Forest plot of the effect of glucocorticoid on PONV on POD1 after TKA**

Supplement: Supplementary file 1 — Supplementary Material 1. [file 12871_2024_2530_MOESM1_ESM.zip › Supplementary figures/Supplementary figure 5.pdf]

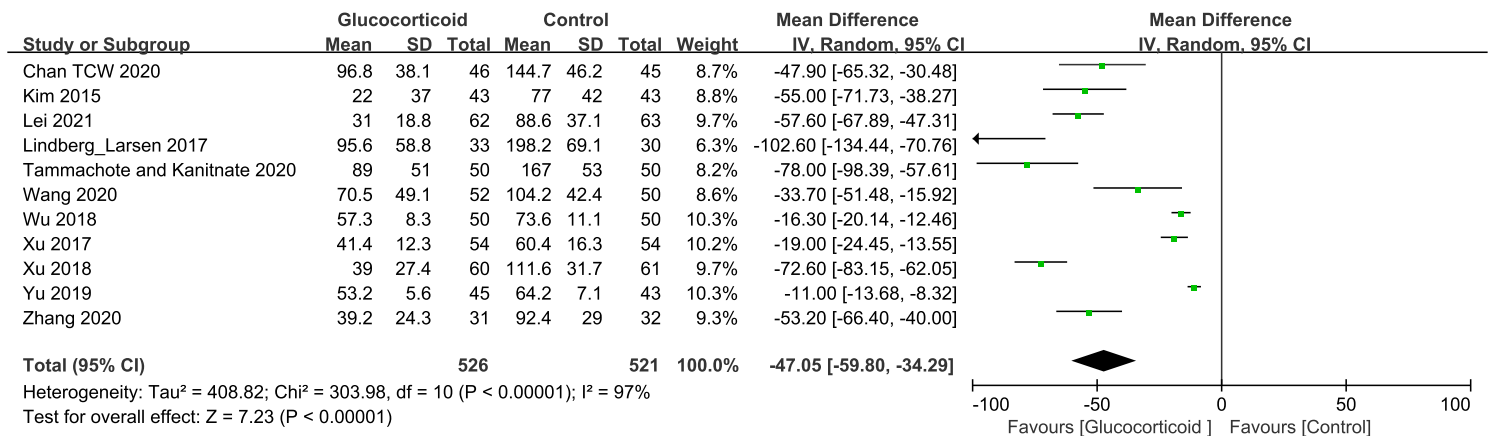

**Figure S2. Forest plot of the effect of glucocorticoid on plasma CRP (mmol/L) on POD2 after TKA**

Supplement: Supplementary file 1 — Supplementary Material 1. [file 12871_2024_2530_MOESM1_ESM.zip › Supplementary figures/Supplementary figure 2.pdf]

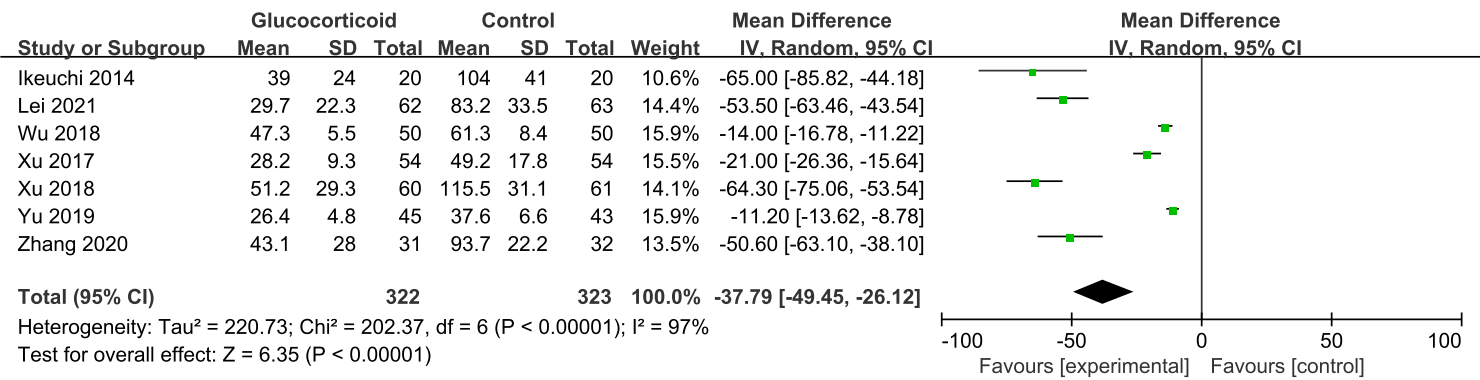

**Figure S3. Forest plot of the effect of glucocorticoid on plasma CRP (mmol/L) on POD3 after TKA**

Supplement: Supplementary file 1 — Supplementary Material 1. [file 12871_2024_2530_MOESM1_ESM.zip › Supplementary figures/Supplementary figure 3.pdf]

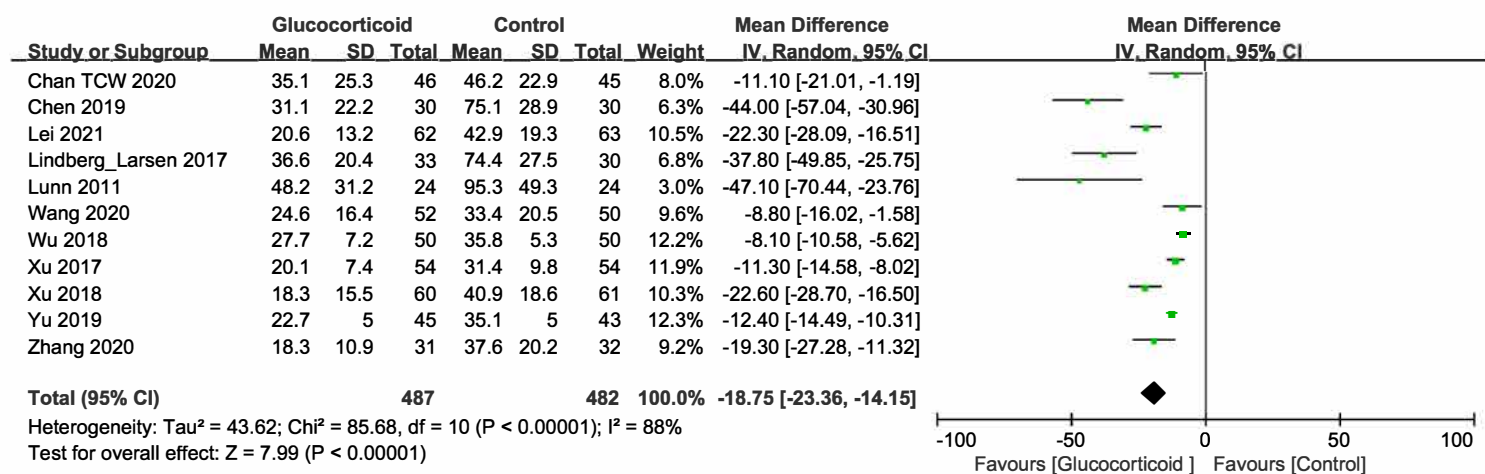

**Figure S1. Forest plot of the effect of glucocorticoid on plasma CRP (mmol/L) on POD1 after TKA**

Supplement: Supplementary file 1 — Supplementary Material 1. [file 12871_2024_2530_MOESM1_ESM.zip › Supplementary figures/Supplementary figure 1.pdf]

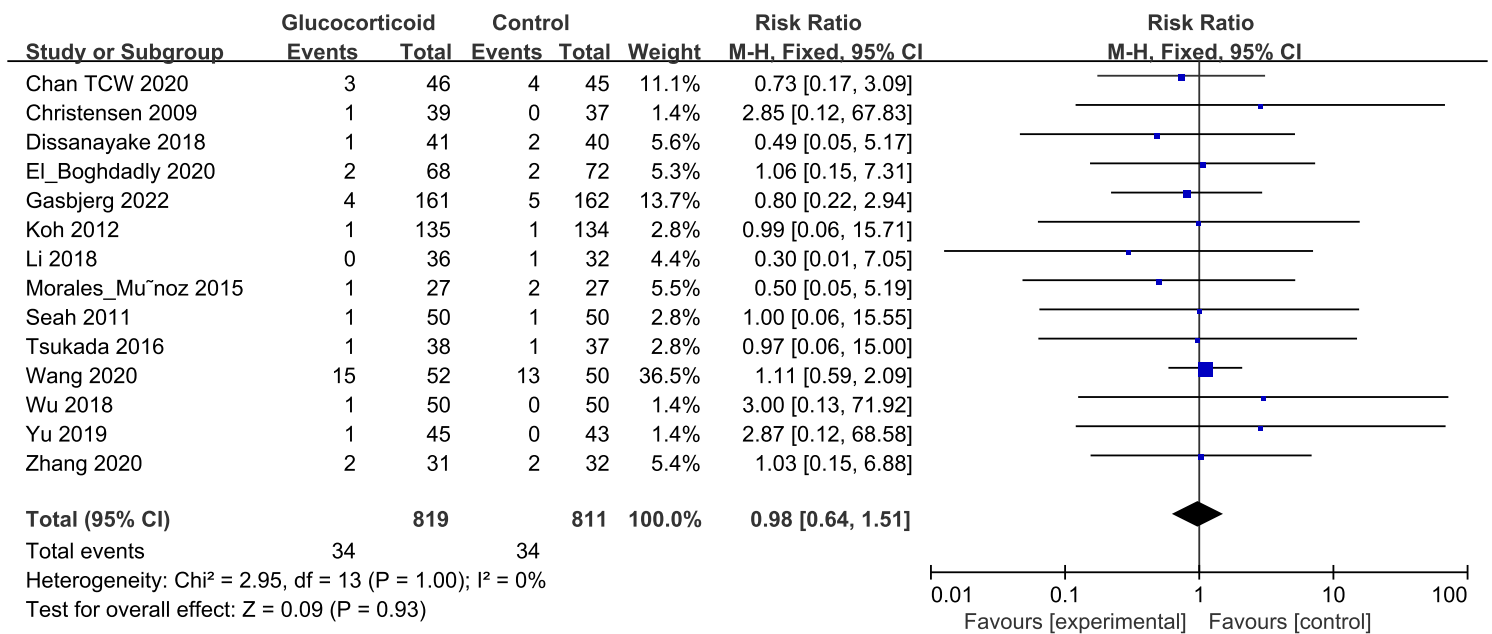

**Figure S10. Forest plot of the effect of glucocorticoid on wound infection after TKA**

Supplement: Supplementary file 1 — Supplementary Material 1. [file 12871_2024_2530_MOESM1_ESM.zip › Supplementary figures/Supplementary figure 10.pdf]

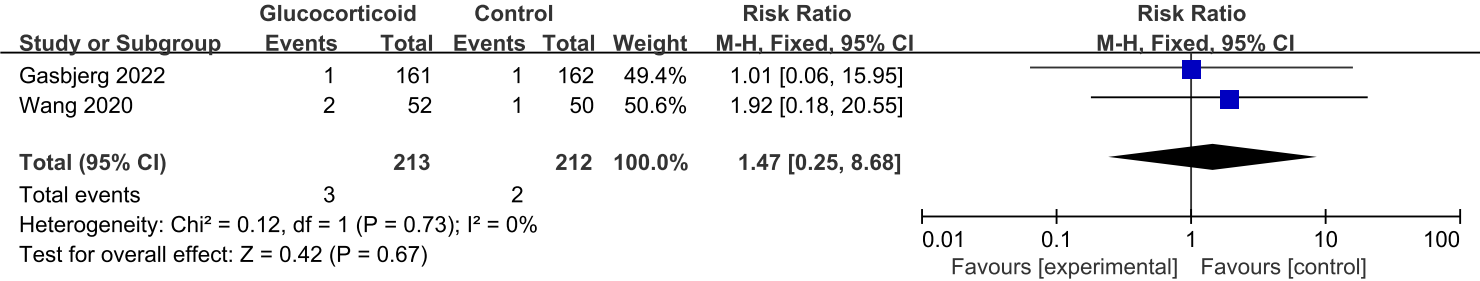

**Figure S11. Forest plot of the effect of glucocorticoid on venous thrombosis after TKA**

Supplement: Supplementary file 1 — Supplementary Material 1. [file 12871_2024_2530_MOESM1_ESM.zip › Supplementary figures/Supplementary figure 11.pdf]

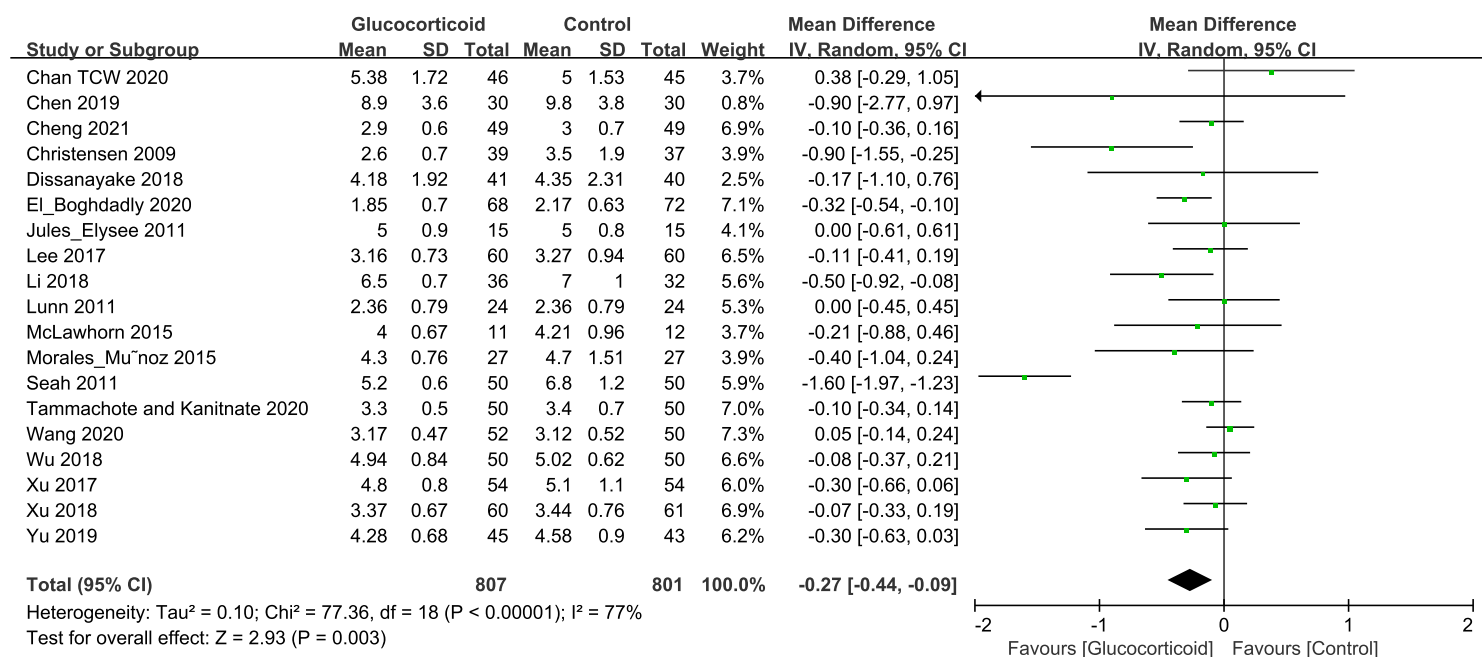

**Figure S12. Forest plot of the effect of glucocorticoid on LOS (days) after TKA**

Supplement: Supplementary file 1 — Supplementary Material 1. [file 12871_2024_2530_MOESM1_ESM.zip › Supplementary figures/Supplementary figure 12.pdf]
